# Biological Activities of Essential Oils: From Plant Chemoecology to Traditional Healing Systems

**DOI:** 10.3390/molecules22010070

**Published:** 2017-01-01

**Authors:** Javad Sharifi-Rad, Antoni Sureda, Gian Carlo Tenore, Maria Daglia, Mehdi Sharifi-Rad, Marco Valussi, Rosa Tundis, Marzieh Sharifi-Rad, Monica R. Loizzo, Adedayo Oluwaseun Ademiluyi, Razieh Sharifi-Rad, Seyed Abdulmajid Ayatollahi, Marcello Iriti

**Affiliations:** 1 Phytochemistry Research Center, Shahid Beheshti University of Medical Sciences, 981991953381 Tehran, Iran; javad.sharifirad@gmail.com (J.S.-R.); razieh.sharifirad@gmail.com (R.S.-R.); majid_ayatollahi@yahoo.com (S.A.A.); 2 Research Group on Community Nutrition and Oxidative Stress, University of Balearic Islands, E-07122 Palma de Mallorca, Spain; tosugo@hotmail.com; 3 CIBER: CB12/03/30038 Fisiopatología de la Obesidad y la Nutrición, CIBERobn, Instituto de Salud Carlos III (ISCIII), E-07122 Palma de Mallorca, Spain; 4 Department of Pharmacy, University of Naples “Federico II”, Via Domenico Montesano, 80131 Napoli, Italy; giancarlo.tenore@unina.it; 5 Department of Drug Sciences, Medicinal Chemistry and Pharmaceutical Technology Section, Pavia University, Viale Taramelli 12, 27100 Pavia, Italy; maria.daglia@unipv.it; 6 Department of Medical Parasitology, Zabol University of Medical Sciences, 61663335 Zabol, Iran; 7 European Herbal and Traditional Medicine Practitioners Association (EHTPA), 25 Lincoln Close, GL20 5TY Tewkesbury, UK; marco.officinalessinia@gmail.com; 8 Department of Pharmacy, Health and Nutritional Sciences, University of Calabria, 87036 Rende (CS), Italy; rosa.tundis@unical.it (R.T.); mr.loizzo@unical.it (M.R.L.); 9 Department of Chemistry, Faculty of Science, University of Zabol, 98615-538 Zabol, Iran; marzieh.sharifirad@gmail.com; 10 Functional Foods, Nutraceuticals and Phytomedicine Unit, Department of Biochemistry, Federal University of Technology, 340001 Akure, Nigeria; ademiluyidayo@yahoo.co.uk; 11 Departament of Biochemical and Molecular Biology, Centre of Natural Sciences and Exatas, Universidade Federal de Santa Maria, 97105-900 Santa Maria, RS, Brazil; 12 Department of Pharmacognosy, School of Pharmacy, Shahid Beheshti University of Medical Sciences, 981991953381 Tehran, Iran; 13 Department of Agricultural and Environmental Sciences, Milan State University, via G. Celoria 2, 20133 Milan, Italy

**Keywords:** isoprenoids, monoterpenes, antimicrobial activity, oral healthcare, traditional healing systems, ethnobotany

## Abstract

Essential oils are complex mixtures of hydrocarbons and their oxygenated derivatives arising from two different isoprenoid pathways. Essential oils are produced by glandular trichomes and other secretory structures, specialized secretory tissues mainly diffused onto the surface of plant organs, particularly flowers and leaves, thus exerting a pivotal ecological role in plant. In addition, essential oils have been used, since ancient times, in many different traditional healing systems all over the world, because of their biological activities. Many preclinical studies have documented antimicrobial, antioxidant, anti-inflammatory and anticancer activities of essential oils in a number of cell and animal models, also elucidating their mechanism of action and pharmacological targets, though the paucity of in human studies limits the potential of essential oils as effective and safe phytotherapeutic agents. More well-designed clinical trials are needed in order to ascertain the real efficacy and safety of these plant products.

## 1. Introduction

In evolutionary terms, plant secondary metabolism greatly contributed to the colonization of terrestrial environments by plant organisms. Indeed, in an ecological context, secondary metabolites relate plants to their ecosystem. Pigments and aromatic compounds confer colour and scent to reproductive organs and fruits, thus attracting pollinators and favouring seed dispersal by animals. However, volatile compounds can also repel phytophagous organisms, including virus and phytoplasma vectors, whereas phytoalexins are broad-spectrum antimicrobial metabolites [1].

In this complex scenario, humans have greatly benefited from plants and their secondary metabolites. During the plant-human coevolution, plants represented (and represent) a nearly unlimited source of food, feed for domesticated animals, fibres for clothes and, not the least, medicaments. Among the vastness of plant products, essential oils deserve particular attention. These are complex mixtures of hydrocarbons and oxygenated hydrocarbons arising from the isoprenoid pathways, mainly consisting in monoterpenes and sesquiterpenes. Essential oils are produced and secreted by glandular trichomes, specialized secretory tissues diffused onto the surface of plant organs, particularly flowers and leaves [2].

This review deals with essential oils from both the plants’ and humans’ perspective, focusing on their pivotal role in plant chemoecology and as source of bioactive phytochemicals and phytotherapeutics for humans. Special emphasis will be put on the uses of essential oils in different traditional healing systems worldwide. Mechanisms of action, and molecular and biochemical targets of essential oils both in prokaryotic and eukaryotic cells will be reviewed, as well as the preclinical and clinical evidences of their efficacy.

## 2. Plant Secondary Metabolites

A long-standing discussion in plant physiology is whether the so-called secondary metabolites (henceforth SMs) are just waste products or they have a role in increasing plant fitness. The co-evolutionary hypothesis, as presented by Fraenkel, Erlich and others since the 1960s [3,4] proposes that plant-animal relationship is a fundamental factor for increasing biodiversity in both insects and plants [5] and that SMs are part of a chemical defense system that enables plants to defend themselves against predators, forcing them into adapting to these chemicals and becoming specialists, in a long-term chemo-evolutionary arm’s race. This hypothesis is not tenable in this early formulation, because it is inconsistent with the fact that amongst SMs there is an unexpectedly large number of closely related molecules or analogs [6] with very low bio-molecular activity [7,8,9]. New hypotheses, such as the “screening” hypothesis [10] and the “silent metabolism” hypothesis [11], have proposed that the adaptive advantage for plants resides not in possessing a specific molecule, but in the availability of many compounds that can be challenged by evolution [10,12]. In fact, it seems that plants with higher chemical diversity show better defenses than those with more limited diversity [13].

One particular group of SMs is the volatile ones, comprising mainly terpenoids, but also phenylpropanoids, polyketides and nitrogen- and sulfur-containing compounds. Although to describe this heterogeneous group of compounds the term “essential oils” has been used, this actually defines the molecules extracted from the plants [14], while to define the volatile SMs as they are in the living organism the term plant volatiles (henceforth PVs) is preferred.

### 2.1. Ecological Role of Biogenic Volatile Organic Compounds

PVs are supposed to mediate the relationship of a plant with abiotic factors such as light, temperature, draught, CO_2_ levels and ozone levels, and biotic factors such as competitors (both interspecific and intraspecific), microbial pathogens, herbivores and other harmful insects, and beneficial animals such as pollinators and seed dispersers [13,15,16]. However, although the evidence for a general ecological role is ample, the specific role of the majority of the molecules involved remains unknown [16].

The chemical defense responses can be described as constitutive or inducible, and as direct or indirect [16]. Constitutive PVs are already present in the organism, they are expressed irrespective of external stimuli, and are mainly made of terpenoids plus shikimate derivatives and polyketides. They seem to have the following functions [5]: reduction of abiotic stress, allelopathy, defense against herbivores, inter-plant signaling, defense against microbial pathogens, attraction of pollinators and seed dispersers.

Constitutive PVs act on herbivores mainly by directly repelling them and/or by inhibiting their feeding. They also act against microbial pathogens by inhibiting their growth via direct antibacterial, antiviral and antifungal activities, and also by repelling viral vectors (such as aphids—although some vectors are actually attracted by PVs) [5].

Inducible PVs are activated by pathogen or herbivore attacks [14]. They have some metabolic costs, but they make the plant phenotypically plastic, and pathogen/insect adaptation more unlikely. They have the following functions [13]: reduction of abiotic stress, defense against herbivores, mainly indirectly but also directly, inter- and intra-plant signaling, defense against microbial pathogens.

More specifically herbivore attack and feeding cause the release of defense signals and defense responses (herbivore-induced plant volatiles, HIPVs). After an herbivore attack, there is an extensive gene expression rearrangement in the attacked plant that seems to be at the origin of HIPVs synthesis [13]. Wounding causes systemin to be cleaved from the precursor prosystemin, and to bind to a SR160 receptor which, in turn, via MAPK and other mechanisms, causes phospholipases to cleave polyunsaturated fatty acids (PUFAs) from the cell membrane. PUFAs are then taken up by plastids and peroxisomes to synthesize, via octadecanoic pathway, jasmonic acid which will act upon the genes in the nucleus of the cell, causing a de novo synthesis of compounds [5,12,17]. Plants react differently to diverse herbivores: they seem capable to recognize different feeding patterns, insect oral secretions and oviposition fluids [13,15,16].

Secreted HIPVs comprise mono- and sesquiterpenes, but also specialized groups of molecules such as green leaf volatiles (henceforth GLVs) and phytohormones such as ethylene, methyl salicylate, jasmonic acid, and others [13,15,16].

GLVs are C_6_ reactive electrophile species, comprising aldehydes such as (*Z*)-3-hexenal and *n*-hexenal, alcohols such as (*Z*)-3-hexenol, and esters such as (*Z*)-3-hexen-1-yl acetate and its *E*-isomers. They derive from cellular membrane C_18_ fatty acids, which are cleaved by LOX/lyase enzymes to give C12 and volatile C6 GLVs, which are released in a matter of minutes after the attack [5,13]. They work by attracting herbivore predators and parasitoids. Thanks to the signaling, these pests can distinguish between damaged and undamaged plants and between plants infested with different herbivore insects. Beyond attracting predators, GLVs can also, together with jasmonic acid, salicylic acid, ethylene and other phytohormones [5], modulate the systemic acquired resistance (SAR) in the same plant and in others, priming them for future attacks [13].

An ideal chronology of plants’ responses to an attack could be thus described: (i) herbivore attack: immediate release of constitutively synthesized PVs upon rupture of storage structures such as glandular trichomes; (ii) from few seconds to few minutes after the attack, wounding causes the release of de novo synthesized, induced PVs (HIPVs) such as GLVs and terpenoids; (iii) continuous damage, oral secretions, oviposition fluids, infection or HIPVs signaling: release, after 12–24 h, usually for the next photoperiod, of induced phytohormones like jasmonic acid, salicylic acid, ethylene, and de novo synthesis of terpenoids and shikimate derivatives.

### 2.2. Plant Volatiles Functions

#### 2.2.1. Allelopathy

*Salvia leucophylla* Greene is an exemplary case of allelopathy and of the role played by monoterpenes (especially 1,8-cineole and camphor in this case) as inhibitors of seed germination and of competition. A revision of the exact modality of *Salvia* PVs release has been proposed by Sakai and Yoshimura [18]: volatilization from living leaves to the atmosphere and subsequently passage to the soil; leaching from living and dead leaves directly to the soil; volatilization from dead leaves to the atmosphere and then passage to the soil.

#### 2.2.2. Adaptation to Abiotic Stresses

PVs synthesis is affected by temperature, light, draught, CO_2_ and ozone levels. Isoprene and monoterpenes increase general thermal tolerance of photosynthesis, protect photosynthetic apparatus and help it to maintain photosynthetic activity under high temperature stress (temperatures above 40 °C) by stabilizing the thylakoid membranes and quenching ROS [19].

#### 2.2.3. Intra-Plant Signaling

HIPVs, especially (*Z*)-3-hexenyl acetate, but also constitutive PVs, can travel from a herbivore-damaged part to an undamaged one, leading to better protection of the latter, probably via activation of defense genes, priming of the tissues and consequent more vigorous response after a real attack. Since plants have a limited vascular connection, and even when it exists, phloem-mediated signaling is slow; HIPVs can be used instead to prime other parts of the plant for a possible attack [5].

#### 2.2.4. Inter-Plant Signaling

Plants (both conspecific and heterospecific) exposed to HIPVs show increased transcription of defense-related genes and altered levels of defense-related metabolites [15]. HIPVs are not the only active compounds, and it has been shown that constitutive PVs from undamaged plants can also induce a defense response [5].

#### 2.2.5. Direct Defense against Herbivores and Pathogens

Direct defense responses comprise PVs that are toxic and repellent; in addition, they can be antinutritional agents and reduce digestibility, growth and reproduction [13]. They target biological systems such as the nervous system, the digestive system, the endocrine organs, and tend to be repellent or toxic for generalists and attractant for the specialists, forcing the latter to use detoxifying mechanisms and therefore reducing their growth and development. They can be constitutive or inducible (HIPVs). The latter are released after herbivore attack; hence they might support the activity of constitutive PVs (acting immediately) by repelling the conspecifics of the attacking insect [13].

#### 2.2.6. Indirect Defense

Indirect defense responses are normally inducible (HIPVs) and comprise volatile compounds that attract, nourish or otherwise favor another organism that reduces herbivore pressure [20]. HIPVs release can attract the natural enemies of the herbivore. They are usually insect-specific, in that their composition varies with the attacking insect, and therefore attracts the specific predators. While there is implicit assumption that these emissions attract predators, there is in fact little field evidence that HIPVs actually reduce herbivore population and increase plant fitness [20]. It has been argued that HIPVs should not be evaluated individually, but as part of a larger signaling network, and it has also been proposed that they might play multiple roles when a plant is attacked by different insects, being repellent for one herbivore species and attractant for another plus its predators and parasitoids [16].

### 2.3. Glandular Trichomes as Secreting Organs

Glandular trichomes (GTs) are modified epidermal hairs containing cells specialized for PVs synthesis and secretion [21]. Noteworthy, other internal secretory structures can also synthetize and secrete PVs. GTs are found on leaves, stems, more rarely on petals, sepals, and petioles in roughly 30% of all vascular plants. They are widespread in eudicots, specifically in Lamiales, Solanales, Asterales, Sapindaceae, Saxifragaceae [21], and especially in Asteraceae, Lamiaceae and Solanaceae [17].

GTs is usually not connected to the vascular system [17], but develops from epidermal cells via periclinal cell divisions [22]. They are usually multicellular, composed of three types of cells: basal, stalk and apical, and they show a sub-cuticular space (henceforth SCS) covered by a toughened cuticle, in which no pore or perforations are present, and which contains both hydrophilic and lipophilic secretions [22]. They have heavily cutinized cell walls of the stalk cells, to protect other tissues from auto-toxicity [17].

They are generally divided in two large subgroups, peltate and capitate GTs. Capitate GTs usually has one basal cell, one to many stalk cells and one to a few apical cells. They usually secrete non- or poorly-volatile SMs stored in a large SCS [17]. Peltate GTs produce the majority of PVs, usually have one basal cell, one stalk cell and up to eight apical cells, and a SCS that stores the PVs [23].

The GTs cells differ from normal plant cells in that they have a very dense cytoplasm with nucleus and nucleolus, no large central vacuole, extensive endoplasmic reticulum (ER), many plastids (amoeboid leucoplasts), relatively few Golgi bodies, abundant mitochondria and numerous plasmodesmata [22].

#### 2.3.1. Biosynthesis of Plant Volatiles in Glandular Trichomes

**Figure 1:**
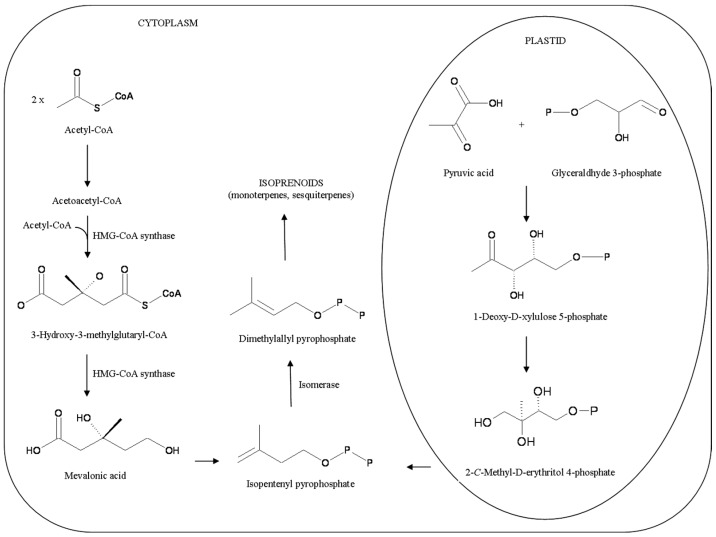
Isoprenoid biosynthetic routes in plant cell: mevalonic pathway, in cytoplasm, and 2-*C*-methyl-d-erythritol-4-phosphate in plastids; isoprenoids synthesis from precursors (isopentenyl pyrophosphate and dimethylallyl pyrophosphate) occurs in cytoplasm.

**Figure 2:**
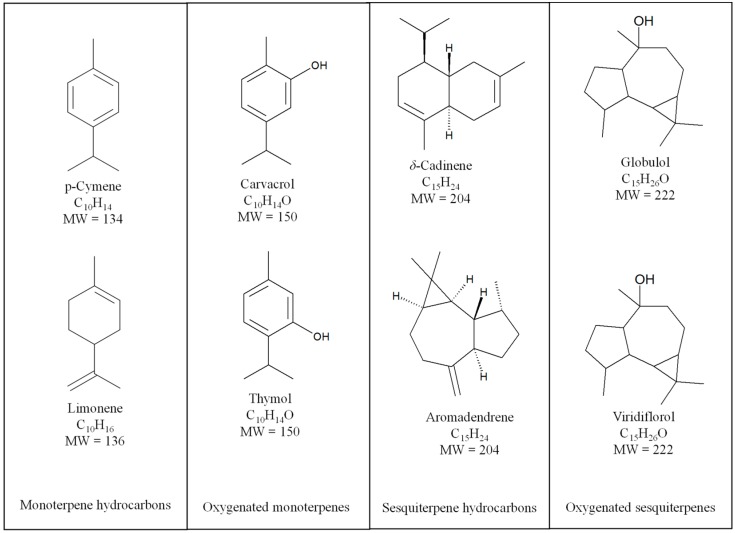
Biogenic volatile compounds produced by plants include monoterpene and sesquiterpene hydrocarbons, as well as oxygenated monoterpenes and sesquiterpenes, also referred to terpenes and terpenoids, respectively; these compounds are the main constituents of essential oils.

There are two main pathways for terpene biosynthesis, and most researchers hold that all steps of this route take place in the secretory cells themselves [24] (Figure 1):

Mevalonic acid (MVA) pathway operates in the cytosol, but sub-cellular details are lacking, perhaps there is an involvement of endoplasmic reticulum (ER) and peroxisomes. It synthesizes sesquiterpene precursors (although in some instances there is a very small production of monoterpenes) (Figure 2).

All the enzymes of the other route, the 2C-methyl-d-erythrytol 4-phosphate (MEP) pathway are located in the plastids. It synthesizes only monoterpene precursors (although in some instances there is a very small production of sesquiterpenes). Although MEP is plastid-located, the enzymes necessary for sesquiterpene synthesis are cytosolic. Hence, MEP-derived intermediates isopentenyl pyrophosphate (IPP) or dimethylallyl pyrophosphate (DMAPP) need to travel from plastids to cytosol. It has been suggested that MEP dominates terpenoid production [25].

The intracellular compartmentalization of terpene biosynthesis is still unclear. It has been observed that at secretory stage GTs show highly developed smooth endoplasmic reticulum (SER), amoeboid leucoplasts, sometimes surrounded by periplastic SER, with many plastid-SER membrane contacts. These plastids showed the greatest changes during development and in relations to secretions [22], correlate strongly with monoterpenes in vivo, and can synthesize monoterpene hydrocarbons in vitro if fed with precursors (IPP and DMAPP) [21].

The close association, in secreting peltate GTs, of plastids, SER (whose surface serves as the location of terpene enzymes) and plasma membrane, the common presence of membrane contact sites (MCSs) between ER and leucoplasts, ER and plasma membrane, and ER and mitochondria suggests that plastids (and maybe SER) have an important role in monoterpene biosynthesis [21].

In fact, IPP and DMAPP seem to originate in the plastids from the MEP pathway, while the stroma of leucoplasts contains the enzymes for the first steps of monoterpene biosynthesis (with or without the involvement of rough ER) [21]. As shown for *Mentha x piperita*, ER, mitochondria and cytosol may be all involved: beginning with the synthesis of (−)-limonene from IPP and DMAPP in leucoplasts, its hydroxylation to (−)-*trans*-isopiperitenol in SER, its conversion to (−)-*trans*-isopiperitenone in mitochondria, and a final creation of many metabolites in cytosol [21]. The next passages are the transfer of PVs (via MCSs) to vacuoles or to ER vesicles, where there could be an amount of processing, and finally transfer to the SCS by exocytosis [22,23].

#### 2.3.2. Ecological Roles of Glandular Trichomes

The primary function of GTs (in particular in the Lamiaceae) is related to defense responses, both constitutive and inducible, against herbivores and pathogens [22], and it has been suggested that the ability to sequester PVs in secretory structures is a critical adaptation in plant-herbivore and plant-pathogen interactions [26].

They can reduce insect movement, protect beneficial phylloplane organisms, deter insects and herbivores, immobilize them, inhibit fungal and bacterial attacks, and mediate allelopathy [27].

GTs can also act, in some species, as detection sensors. The disruption of GTs by walking insects can induce a defensive readiness that allows plants to respond more quickly. It triggers release of JA, JA signaling and induction of defense genes within 3–24 h. Thus, any plant can be primed by walking, exploring or ovipositioning insects to a state of intermediate alarm. Although this priming is non-selective (triggered also by natural enemies of the herbivore), it is energetically advantageous because it allows an intermediate (less expensive) step between no alarm and full-fledged alarm [28].

In certain cases, SMs in GTs can be transported via the stalk to distal plant tissues to protect them, as in the case of pyrethrins produced in fruit GTs that are transferred to the seeds which, being hairless, cannot secrete their own chemical defenses [17].

They also have non-defensive roles, such as temperature regulation, light reflectance, decreased water loss (via light reflection), reduced mechanical abrasion, reduced leaf humidity, reduced photosynthesis (via light reflection), attraction of pollinators and seed dispersal [17].

## 3. Mechanisms of Essential Oil Cytotoxicity

Essential oils are a complex mixture of molecules, which generally contains more than 20 different components of low molecular weight with very variable concentrations. In general, monoterpenes and sesquiterpenes are the main components of essential oils, though diterpenes and phenylpropanoids can be present to a different extent. Many of these molecules are found in low concentrations, while few of them are the main components that can represent up to 70% of total oil and will be the main responsible for the biological effects of the oil [29,30]. Until now, more than 3000 essential oils have been described, of which about one tenth are relevant for pharmaceutical, nutritional or cosmetic industries. Several essential oils have a strong interest in research for their cytotoxic capacity. Great efforts are performed in order to investigate the potential therapeutic effects of oils against several diseases especially those characterized by excessive cell growth and proliferation such as cancer or bacterial infections [31,32]. The main mechanisms that mediate the cytotoxic effects of essential oils include the induction of cell death by activation of apoptosis and/or necrosis processes, cell cycle arrest, and loss of function of essential organelles. Several of these effects are attributable to the lipophilic nature and low molecular weight of the main components that comprise essential oils which allow them to cross cell membranes, alter membrane composition and increase membrane fluidity, leading to leakage of ions and cytoplasmic molecules. Altering membranes lead to reduced ATP production, alteration of the pH gradient, and loss of mitochondrial potential that can result to the cell death. In addition, some essential oils may also act as pro-oxidant elements which can alter cellular redox state and also compromise cellular survival.

The cytotoxic properties of essential oils result from the complex interaction between the different classes of compounds such as phenols, aldehydes, ketones, alcohols, esters, ethers or hydrocarbons [29,32,33]. In addition, in some cases, the cytotoxic activity are closely related to few of the main components of the oils and, in this way, it has been reported that some of these isolated compounds exert considerable cytotoxic properties when have been tested individually [33,34,35]. However, the wide variation in the chemical profile of essential oils means a great diversity in the mechanisms of action and molecular targets. Furthermore, because these oils consist of a wide variety of compounds, each compound can modulate or alter the effects of other ones.

### 3.1. Molecular And Biochemical Targets in Prokaryotic Cells

Plants produce a wide variety of secondary metabolites many of which play a role in plant protection against predators and microbes potentially pathogenic derived from their cytotoxic properties or reduced palatability against herbivores. In addition, the increasingly selection of bacteria resistant to many antibiotics has led to research into the use of essential oils as potential alternatives. The presence of components with phenolic structures or aldehydes, such as thymol, carvacrol, eugenol, cinnamaldehyde and citral, were greatly active against microorganisms [36,37]. These compounds were very active despite their relatively low capability to dissolve in water. The importance of the hydroxyl group in the phenolic structure was confirmed by the higher antimicrobial activity when carvacrol or eugenol was compared to their respective methyl ether [36,38]. In this way, the antimicrobial activity of members of the genus *Thymus* and *Origanum*, *Ocimum basilicum* L. and *Cinnamomum zeylanicum* Breyne are mainly associated to the presence of thymol, carvacrol, eugenol and cinnamaldehyde, respectively [39,40,41]. However, most of the studies were simply focused on investigating the antibacterial activities of essential oils and/or some of the major compounds rather than exploring the mechanisms of action involved in their bioactivity.

The cytotoxic effects of essential oils are primarily made through disrupting the structure of membranes, leading to bacterial cell permeabilization. As a result of membrane permeabilization, all other cellular functions including membrane potential, efflux pump activity or respiratory activity are also compromised [37,42,43]. Moreover, it has been evidenced, using flow cytometry experiments, that the mode of action mediated by bacterial cell permeabilization is similar in both Gram-positive and -negative bacteria [44]. The ability to maintain the membrane potential and pH gradient is necessary for cell survival, and a decrease in these parameters is indicative of significant damage to the cell membrane [45]. The leakage of cellular components into the extracellular space such as potassium, ATP or DNA (260 nm-absorbing cell material) is also an indicator for an increase in membrane permeability and loss of cell viability. In addition, uptake of substances such as propidium iodide or *N*-phenyl-l-napthylamine indicates that formation of unregulated pores in the membrane and an increased probability of cell death. Finally, cell-to-cell communication among bacteria (quorum sensing), used to control group behaviors, including virulence factor production or biofilm formation, is another central target that could help to reduce antimicrobial resistance [46].

Diverse studies reported the capability of essential oils to alter the membrane structure and permeability observed with electron microscopy and, in most of cases, also evidenced by the release of diverse elements outside the cell. Lemongrass essential oil caused discernible cell membrane alterations and formed electron-dense inclusions detected with electron microscopy to planktonic and sessile growth of a sulfate reducing bacterium [47]. The leaf essential oil of *Forsythia koreana* Nakai induced changes in cell wall morphology, cell wall lysis, and pore formation of *Escherichia coli* and *Listeria monocytogenes* [48]. In this study, the release of potassium ions and 260 nm-absorbing cell material was also increased in the presence of the essential oils. Similar structural alterations were evidenced using the essential oil of *Pimenta pseudocaryophyllus* (Gomes) Landrum against the main bacteria responsible for bad perspiration odor (*Staphylococcus epidermidis* or *Proteus hauseri*) and *Cinnamomum longepaniculatum* (Gamble) N. Chao ex H. W. Li leaf essential oil against *Staphylococcus aureus*, *E. coli* and *Salmonella enteritidis* [49,50]. The essential oil of *Melaleuca alternifolia* L. (tea tree) disrupted the permeability barrier of *E. coli* and *S. aureus* membranes leading to the loss of chemiosmotic control [51]. The treatment with tea trea oil caused a potassium ion leakage, more evident in the case of *E. coli*, but also inhibited the respiration and increased the permeability of bacterial membranes as determined by uptake of propidium iodide. *Origanum compactum* Benth (oregano) and *Cinnamomum verum* J. Presl. essential oils caused potassium leakage and uptake of propidium iodide in both *Pseudomonas aeruginosa* and *S. aureus* associated with loss of membrane permeability and structural alterations [52,53]. *Ginkgo biloba* L. leaf essential oil was also investigated against foodborne pathogenic bacteria [54]. The results evidenced that *G. biloba* oil induced considerable morphological alterations on the cell wall of diverse foodborne pathogenic bacteria which was also related to release of extracellular ATP, increase of 260 nm-absorbing materials and leakage of potassium ions. The essential oil from edible seaweed, *Enteromorpha linza* (L.) J. Agardh, was also highly active against the foodborne pathogenic bacteria *Bacillus cereus* and *S. aureus*, inducing significant increase in leakage of 260 nm-absorbing materials and potassium ions from the cell membrane and loss of high salt tolerance [55]. An interesting study evaluated the effects of an essential oil from *Thymus daenensis* Čelak. formulated as a water-dispersible nanoemulsion in order to facilitate the access of the essential oil into the bacterial cell [56]. The nanoemulsion amplified the antibacterial activity of the essential oil that was evidenced by enhanced potassium and nucleic acid leakage. In another study, citrus oil from orange (*Citrus sinensis* (L.) Osbeck.) and bergamot (*Citrus bergamia* Risso & Poit.) (1:1 *v*/*v*) was tested against *Enterococcus faecium* and *Enterococcus faecalis* [57]. The results indicated that the oil was able to induce large pore formation as it was evidenced by a significant uptake of *N*-phenyl-1-naphthylamine. Moreover, a decrease in intracellular pH, in membrane potential and a reduced ATP synthesis were also reported. Essential oils from *Satureja hortensis* L. and *Salvia fruticosa* Mill. altered the outer membrane permeability of *Fusobacterium nucleatum*, key bacteria in oral biofilms [58]. Membrane permeability, tested by measuring the *N*-phenyl-1-naphthylamine uptake, was significantly increased by the treatment with both essential oils. The treatment of *E. coli* and *L. monocytogenes* with Spanish oregano (*Coridothymus capitatus* Rchb. f.), Chinese cinnamon (*Cinnamomum cassia* (L.) D. Don.), and savory (*Satureja montana* L.) essential oils induced the depletion of the intracellular ATP concentration, reduced intracellular pH, and evident damage to cell membranes [59].

The effects of essential oils were also investigated against quorum sensing. An interesting work investigated the inhibitory effects of 21 essential oils against quorum sensing in *Chromobacterium violaceum* and *P. aeruginosa* [60]. Clove oil demonstrated the most anti-quorum sensing activity by inhibiting violacein pigment production in *C. violaceum* and swarming motility in *P. aeruginosa*, followed by cinnamon, lavender, and peppermint oils. A similar study performed by Szabó et al. [61] using the same *C. violaceum* as sensor strain evidenced that rose, geranium, lavender and rosemary oils were highly potent quorum sensing inhibitors. On the other hand, eucalyptus and citrus oils reported moderate effects, and chamomile, orange and juniper oils were ineffective. The oregano essential oil has been also reported to exert inhibitory quorum sensing activity using *C. violaceum* as bacterial model [62]. Peppermint oil strongly interfered with acyl homoserine lactone regulated virulence factors and biofilm formation in *P. aeruginosa* and *Aeromonas hydrophila*, indicating a broad-spectrum of activities [63]. Moreover, oregano essential oil (*Origanum heracleoticum* L.) also inhibited the expression of virulence-associated genes in enterohaemorrhagic strain of *E. coli* [64]. In another study, pyocyanin, pyoverdine, elastase and biofilm production was decreased in *P. aeruginosa* when treated with ferula oil (*Ferula asafetida* H.Karst), whereas pyoverdine and elastase production, but not pyocyanin and biofilm production were decreased after dorema (*Dorema aucheri* Boiss.) treatment [65].

Finally, it is interesting to report that some investigations evidenced that essential oils can be useful against multidrug resistant bacteria. The group of Yap evidenced in multidrug resistant *E. coli* strain that cinnamon bark essential oil as well as lavender essential oil induced irreversible membrane damage and also inhibited quorum sensing evidenced by reduced production of bioluminescence [66,67]. The most polar fraction obtained from *Cistus ladaniferus* Gouan ex Steud essential oil, which was mainly constituted by mono- and sesquiterpene alcohols, induced cell wall distortion with an outer cytoplasmic membrane detachment in a multidrug resistant strain of *Enterobacter aerogenes* [68]. *Eucalyptus camaldulensis* Dehnh. and *Myrtus communis* L. essential oils were tested against multidrug resistant *Acinetobacter baumannii* wound isolates. Both essential oils evidenced antibacterial effects when administered alone, but also reported synergistic effects when combined with antibiotics [69,70].

### 3.2. Molecular and Biochemical Targets in Eukaryotic Cells

#### 3.2.1. Anti-Cancer Activity

At first, most of the essential oils were investigated for their antioxidant and anti-inflammatory properties and, consequently, for their potential use in the treatment of inflammatory diseases. Moreover, essential oils could also exert anticancer effects because there is a direct relation between the production of reactive oxygen species (ROS) and oxidative and inflammatory states that can lead to cancer [71,72]. On one hand, an overproduction of ROS is associated with chronic inflammation and can also induce DNA damage increasing the mutation rate and the probability that cells undergo oncogenic transformation [73]. On the other hand, it is well established that ROS are able to modulate redox-mediated signalling pathways which can lead to tumor development. Until to date, there are more than five hundred published articles focusing on anticancer activity of essential oils [33]. Drugs used in the treatment of cancer have as a primary aim the induction of apoptosis or cell cycle arrest in cancer cells. Thus, the essential oils that are capable of inducing apoptosis in cancer cells may be potential resources for coping with cancer. In addition to the apoptosis induction, other mechanisms which help in cancer treatment are the activation of the detoxification and DNA repair systems, and the inhibition of metastasis and angiogenesis [74,75]. Essential oils exert anti-proliferative effects in diverse cancer cell models through diverse pathways.

Apoptosis is a well-defined form of programmed cell death to ensure homeostasis which can be triggered by endogenous or exogenous signals. An abnormality in apoptosis process can origin various types of diseases such as cancer or autoimmune diseases. Due to the high heterogeneous composition of essential oils together with the wide types of cancers, it is quite difficult to define an exclusive mechanism of action. Many studies have reported the anticancer activity of essential oils and some of their isolated components against several cancers such as glioblastoma, melanoma, leukaemia, bone, breast, lung, ovary, pancreas and prostate cancers, among others (see Bayala et al., 2014 for review). In most of the studies, apoptotic markers including cytoskeletal alterations, plasma membrane damage, mitochondrial dysfunction, DNA fragmentation, caspase-3 activation, and cleavage of pro-survival proteins have been reported [76,77,78,79].

One of the mechanisms by which essential oils can induce apoptosis is through increased generation of ROS. The *Abies balsamea* (L.) Mill. (balsam fir oil) essential oil was tested against solid tumour cell lines (MCF-7, PC-3, A-549, DLD-1, M4BEU and CT-26) and reported significant cytotoxicity in all these cell lines [80]. The treatment with the essential oil depleted cellular reduced glutathione (GSH) content and increased ROS production in a dose- and time-dependent manner. The volatile extract from dried pericarp of *Zanthoxylum schinifolium* Siebold & Zucc. also induced apoptotic death in HepG2 human hepatoma cells and significantly increased ROS production [81]. However, no effect was reported in caspase-3 activity, suggesting that the extract-induced apoptosis of hepatoma cells is caspase-3 independent. The effects of the essential oil from rosewood *Aniba rosaeodora* Ducke were investigated on the human epidermoid carcinoma cell line A431 and on immortal HaCaT cells [82]. The treatment reported evident cytotoxicity in both cell types triggered by the production of ROS, with depolarization of the mitochondrial membrane and caspase-dependent cell death. In another assay, the essential oil from the leaf of *Pinus densiflora* Siebold & Zucc. inhibited the proliferation and survival and induced apoptosis in YD-8 human oral squamous cell carcinoma cells [83]. The treatment with oil led to generation of ROS which, in turn, activated caspase-9 activity, the DNA repair enzyme poly(ADP-ribose) polymerase (PARP) cleavage, down-regulation of the anti-apoptotic protein B-cell lymphoma 2 (Bcl-2), and phosphorylation of extracellular signal-regulated kinase (ERK)-1/2 and c-Jun *N*-terminal kinase (JNK)-1/2. *Melissa officinalis* L. essential oil was also tested in glioblastoma multiforme cells showing a significant induced apoptosis as it was evidenced by DNA fragmentation and caspase-3 activation [84]. The cytotoxicity was mediated by ROS because antioxidants prevented cell death. *C. bergamia* essential oil induced apoptotic and necrotic cell death in human neuroblastoma SH-SY5Y cells [85]. The increased ROS generation was responsible for the activation of tumour suppressor protein p53 by phosphorylation, increased levels of the pro-apoptotic Bax and reduced Bcl-2 and a reduced phosphorylation of p38 and ERK-1/2.

Another target for essential oils is the protein kinase B, also known as Akt, which regulates p53. Moreover, the Akt pathway is found to be activated in early stages of diverse cancers, and activation of Akt signalling protects cancer cells from tamoxifen-induced apoptosis [86,87]. *Boswellia sacra* Flueck. essential oil induced tumor cell-specific apoptosis in several human breast cancer cells with significant fragmented genomic DNA, caspase-3 activation and cleavage of PARP [88]. *B. sacra* essential oil suppressed Akt, reducing the levels of phospho-Akt (Ser473), and ERK1/2 activation in human breast cancer cell lines except to MDA-MB-231 cells. In a further research from the same group, *B. sacra* essential oil also induced apoptosis in pancreatic cancer cells, but, in this case, it was associated with a transient activation of PI3K/Akt and ERK1/2 pathways [89]. This differential response makes necessary additional investigations to clarify the biological significance of these opposite behaviour. Volatile oil from *Litsea cubeba* Pers. seeds induced apoptosis and cell cycle arrest in human A549 non-small cell lung carcinoma cells [90]. The treatment with the oil dephosphorylated Akt and subsequently induced the overexpression of p53 and enhanced Bax levels allowing the release of mitochondrial cytochrome c and the activation of caspases. Similar results were obtained using *Curcuma zedoaria* Roxb. essential oil in the same non-small cell lung carcinoma cells [91]. Another study evidenced that *Monarda citriodora* Cerv. ex Lag. activated apoptosis in human promyelocytic leukemia HL-60 cells by means of disruption of the phosphatidylinositol 3-kinase (PI3K)/AKT/mTOR signaling cascade [92]. The anti-cancer effects of the essential oil of *Pinus koraiensis* Siebold & Zucc. were investigated in EOPK in HCT116 colorectal cancer cells [93]. The essential oil significantly reduced the proliferation and migration of the colorectal cancer cells and suppressed the expression of PAK1, a central node for various oncogenic signalling pathways, which in turn reduced the phosphorylation of Akt and ERK.

Mitogen-activated protein kinases (MAPKs) are also essential targets for the essential oils as it was shown in some of data mentioned above. Diverse MAPKs including JNK, ERK and p38 kinases are involved in the apoptosis process in cancer cells. Essential oils can induce apoptosis through phosphorylated MAPKs, a process that can be favoured by ROS production [94]. *Artemisia capillaris* Thunb. essential oil exerted cytotoxicity in human oral epidermoid carcinoma cells [95]. The results suggest the participation of the p38/nuclear factor-kappaβ (NF-κβ) and JNK/Bcl-2-mediated pathways as well as caspase activation in the mechanism of cell death. In the study by Chen et al. [91], apoptosis induced by *C. zedoaria* essential oil in non-small cell lung carcinoma cells in addition to Akt was also mediated by ERK1/2, JNK1/2 and p38. *Thymus vulgaris* L. essential oil cytotoxicity was investigated towards head and neck squamous cell carcinoma UMSCC1 cell line [96]. The most significantly regulated pathways by thyme essential oil determined using microarray hybridization were *N*-glycan biosynthesis, interferon and ERK-5 signalling pathways.

Nuclear factors play an important role in the development of cancer and may also be affected by essential oils so they are an interesting target to be investigated. NF-κβ plays a central role in the regulation of apoptosis, oncogenesis and inflammation and is associated with cancer when over-expressed [97]. In this way, inhibition of NF-κβ could be a useful strategy for cancer therapy, although in some models it seems to facilitate tumour development [98]. The essential oil from a lemon grass variety of *Cymbopogon flexuosus* (Nees ex Steud.) W.Watson induced apoptosis in a dose-dependent manner as evidenced by increased annexin V binding, DNA laddering and apoptotic bodies in HL-60 cells [99]. The oil treatment decreased the expression of nuclear NF-κβ and, consequently, could inhibit its translocation to nucleus. The essential oil of *Pogostemon cablin* (Blanco) Benth. (patchouli) exerts anti-cancer activity against diverse human colorectal cancer cells by inducing apoptosis and decreasing cell growth [100]. The proposed mechanism of action includes the inhibition of histone deacetylase 2 (HDAC2) expression and activity and subsequent downregulation of c-myc oncogene and activation of NF-κβ pathway through an increase of nuclear translocation of p65. *Curcuma wenyujin* Y.H.Chen & C.Ling extract inhibited tumour growth in human cervical cancer HeLa cells through blockade of cell cycle progression at G1 phase and apoptosis [101]. The treatment decreased the expression of p65 subunit of NF-κβ and diminished the phosphorylation of Ikβα leading to downregulation of the NF-κβ pathway. Water soluble extract of *Cinnamomum cassia* (L.) J. Presl inhibited tumor cell proliferation and induced cell death by activating pro-apoptotic molecules and inhibiting NF-κβ and activator protein-1 (AP1, a nuclear factor able to induce transcription of genes involved in cell proliferation, apoptosis and metastasis) activities and their downstream genes such as Bcl-2, BcL-xL and survivin in a mouse melanoma model [102].

Finally, mitochondrion is another key target for essential oils as this organelle can initiate apoptotic processes. The apoptotic effects of *Cryptomeria japonica* (Thunb. ex L.f.) D. Don on human oral epidermoid carcinoma cells could be also mediated by mitochondrial stress and activation of caspases [103]. In fact, the oil treatment increased the mitochondrial level of Bax and decreased Bcl-2, inducing the release of cytochrome c into the cytosol. In another study, it was observed that the treatment with *C. wenyujin* activated the mitochondrial apoptotic pathway in human cervical cancer HeLa cells as it was evidenced by a decrease in myeloid cell leukemia sequence 1 (Mcl-1) and Bcl-xL levels, leading to mitochondrial membrane potential loss and caspase activation [101]. The cytotoxic effects of *Artemisia vulgaris* L. essential oil against HL-60 cells is mediated by a mitochondria-dependent apoptosis [79]. The essential oil significantly altered the mitochondrial transmembrane potential, increased the release of cytochrome c, and disrupted the expression of some members of the Bcl-2 family. The effects of *Cephalotaxus griffithii* Hook. f. needle essential oil on human cervical cancer cells (HeLa, ME-180 and SiHa) evidenced a mitochondria-initiated apoptosis [104]. The essential oil increased mitochondrial membrane depolarisation and enhanced the expression of caspases and PARP cleavage.

#### 3.2.2. Antifungal Activity

Natural products with antifungal properties are also an interesting new therapeutic alternative to the synthetic drugs. These products also become more important by the fact that, similar to bacteria, fungal drug-resistant strains are increasing rapidly [105]. Many studies have demonstrated the effectiveness of essential oils against fungal species, although only few of them have investigated the underlying mechanisms of action [106]. For example, Soylu et al. [107,108] tested several essential oils obtained from aromatic plants including oregano (*Origanum syriacum* var. *bevanii* (Holmes) letsw.), thyme (*Thymbra spicata* L. subsp. s*picata*), lavender (*Lavandula stoechas* L. subsp. *stoechas*), rosemary (*Rosmarinus officinalis* L.), fennel (*Foeniculum vulgare* Mill.) and laurel (*Laurus nobilis* L.) against tomato late blight disease agent *Phytophthora infestans* and tomato grey mould disease agent *Botrytis cinerea*. The treatment with the essential oils provoked the loss of integrity of the cell wall and plasma membrane permeability with important morphological alterations in hyphae. The antifungal activity of phenolic-rich *Lavandula multifida* L. essential oil has been evidenced against *Candida albicans* [109]. The apoptosis induction was evidenced by inhibition of filamentation, cytoplasmic membrane disruption and propidium iodide staining. In another study, Dias Ferreira et al. [110] showed that *Curcuma longa* L. was cytotoxic for *Aspergillus flavus*, and inhibited aflatoxin production. Analysis with scanning electron microscopy reported significant damage to hyphae membranes and conidiophores in *A. flavus* exposed to the essential oil. The antifungal effects of *Thymus eriocalyx* (Ronniger) Jalas and *Thymus* x*-porlock* essential oils were studied in *Aspergillus niger*. Transmission electron microscopy reported that *A. niger* exposed to essential oils reported irreversible damage to cell wall, cell membrane and diverse cellular organelles. *Matricaria chamomilla* L. flower essential oil was probed against *A. niger* growth and ultrastructure [111]. The results reported an evident disruption of cytoplasmic membranes and intracellular organelles, detachment of plasma membrane from the cell wall, and complete disorganization of hyphal compartments. The authors suggested that the morphological alterations could be a consequence of increased cell permeability of the fungal plasma membrane.

In addition to ultrastructural alterations, some studies investigated the mechanisms underlying the pro-apoptotic effects of essential oils. In a study, *Cinnamomum jensenianum* Hand–Mazz essential oil showed significant alterations in plasma membrane, fibrillar layer, and cytoplasm of *A. flavus* [112]. Mitochondria also suffered a wide disruption of the internal structure with a decrease in the mitochondrial cristae. Moreover, the essential oil caused a substantial reduction in the ergosterol quantity in the plasma membrane. Ergosterol is a specific compound of fungi being the main sterol of the cell membrane and also plays an important role in maintaining the integrity and the function of the fungal cell [113]. *Coriaria nepalensis* Wall. essential oil cytotoxicity was examined against fluconazole-sensitive and -resistant *Candida* isolates [114]. The essential oil was effective against all *Candida* isolates by disrupting membrane integrity and also inhibiting ergosterol biosynthesis. The treatment with the essential oil from *Ocimum sanctum* L. induced significant cytotoxicity in *C. albicans* cells [115]. This effect was evidenced by complete ergosterol depletion and membrane disintegration, DNA fragmentation, increased externalization of membrane phosphatidylserine and reduced cytochrome c oxidase activity. *Coriandrum sativum* L. essential oil was also reported to bind to membrane ergosterol, increasing ionic permeability and inducing membrane damage which leads to cell death in different *Candida* strains [116]. In addition, the *C. sativum* essential oil decreased the proteolytic activity of *C. albicans*. Similar results were reported when treating *Candida* strains (*C. albicans*, *C. tropicalis* and *C. glabrata*) with *Mentha piperita* L. essential oil [117]. Exposed cells showed a highly decrease in the ergosterol content, cell membrane disruption, and morphological alterations.

Induction of ROS overproduction and oxidative stress has been also reported to mediate the cytotoxic effects of essential oils. *Anethum graveolens* L. seed essential oil was reported to induce apoptosis in an human pathogen *C. albicans* strain as it was clearly evidenced by a decrease in ATPase activity, chromatin condensation, DNA fragmentation, and phosphatidylserine exposure, cytochrome c release and metacaspase activation [118]. In this study, l-cysteine was able to prevent apoptosis indicating that ROS participated in the essential oil-induced apoptosis. In an investigation by Ferreira et al. [119], the essential oil of *M. piperita* cytotoxic to the yeast *Saccharomyces cerevisiae* was associated with increased levels of intracellular ROS, mitochondrial destruction and chromatin condensation, without loss of the plasma membrane integrity.

Another point to take into account is the production of mycotoxins which can be toxic for humans. Aflatoxins are very harmful fungal toxins and, consequently, the potential effects of natural compounds against toxin production are of great interest [106]. In accordance, the *Ephedra major* Host essential oil reduced growth and aflatoxin production by *Aspergillus parasiticus* [120]. The essential oil extracted from *Chenopodium ambrosioides* L. was cytotoxic against a broad number of fungi and also inhibited the aflatoxin B1 production by the aflatoxigenic strain of *A. flavus* [121]. The essential oils from *T. eriocalyx* and *Thymus* x*-porlock* significantly inhibited *A. parasiticus* growth, aflatoxin production and induced irreversible damage in cell membranes [122]. Antifungal and aflatoxin suppressive effects were also reported in *A. flavus* after treatment with diverse essential oils such as *Piper betle* L. var. *magahi* [123,124,125]. In fact, it was evidenced that *Zataria multiflora* Boiss. essential oil reduced growth and aflatoxin production in *A. parasiticus* [126]. The inhibitory effects of *Z. multiflora* oil on toxin production were associated to an inhibition of the genes of aflatoxin biosynthesis pathway.

#### 3.2.3. Antiparasitic Activity

Plant essential oils can be used as alternatives against endo- and ectoparasites. In this way, *Plasmodium falciparum* and *Leishmania donovani* are protozoan parasites which are becoming resistant to conventional drugs, and this leads to an increase of the mortality and morbidity rates [127,128]. In addition, the control of ectoparasites in veterinary is of great importance because of the development of insecticide resistance [129]. Some authors investigated the potential anti-parasitic effects of essential oils against diverse types of parasites such as protozoa, helminths and arthropods; however, the molecular mechanisms of action are poorly investigated.

The antiparasitic activity *Lavandula angustifolia* Mill. and *Lavandula* x *intermedia* essential oils were assayed against the human protozoal pathogens *Giardia duodenalis* and *Trichomonas vaginalis* and also against the fish pathogen *Hexamita inflata* [130]. Both essential oils completely eliminated all three protozoa in the in vitro assay. The antileishmanial activity of essential oil from *C. ambrosioides* evidenced significant cytotoxic effects against intracellular amastigote form [131,132]. The essential oil obtained from *Piper cubeba* L.f. was active against *Schistosoma mansoni* [133]. The essential oils of four *Cymbopogon* species, *C. citratus* (DC) Stapf., *C. giganteus* Chiov., *C. nardus* (L.) Rendle and *C. schoenantus* (L.) Spreng. were cytotoxic when tested against *Trypanosoma brucei brucei* and *Plasmodium falciparum* [134]. The same authors also reported antitrypanosomal and antiplasmodial activities of essential oils from *Ocimum gratissimum* L. [135]. In another assay, *Artemisia indica* Willd. oil showed in vitro antimalarial activity, in addition to potential malaria prophylactic effect [136]. The essential oil obtained from *Artemisia absinthium* L. showed toxic activity on two parasitic protozoa *Trypanosoma cruzi* and *Trichomonas vaginalis* [137]. Respect to helminths, the essential oils obtained from *Tetradenia riparia* leaves and *Foeniculum vulgare* Mill. decreased the number of eggs produced and the percentage of developed eggs in *Schistosoma mansoni* [138].

Diverse studies also reported evidences of cytotoxicity against ectoparasites such as ticks and mites. The toxicity of *Hesperozygis ringens* (Benth.) Epling. essential oil was tested on engorged females and larvae of the cattle tick *Riphicephalus (Boophilus) microplus* [139]. Another study also tested 11 essential oils from Brazil on reproductive efficiency and lethality of the cattle tick *R. microplus* [140]. All essential oils tested showed efficacy against the cattle tick, being *C. longa* and the members of the *Lippia* genus the most effective and the *Croton* genus the worst. Essential oils were also effective against host-seeking nymphs of the lone star tick, *Amblyomma americanum*, being oregano essential oil the most effective [141]. The acaricidal activities of *Chrysopogon zizanioides* (L.) Roberty essential oils were assayed on *Amblyomma cajennense* and *Rhipicephalus microplus* (Acari: Ixodidae), and promising cyototoxic effects were documented [142]. Finally, the cytotoxic effects were investigated in BALB/c mice infected with *Leishmania amazonensis* and treated with *C ambrosioides* essential oil for 15 days [143]. The treatment with the oil significantly reduced the size of cutaneous lesions. *C. ambrosioides* showed higher toxic activity compared with animals treated with the conventional drugs glucantime, amphotericin B, and pentamidine.

The molecular mechanisms associated to the antiparasitic effects of essential oils were investigated using *Artemisia annua* L. leaf essential oil against visceral Leishmaniasis induced by *Leishmania donovani* [144]. The cytotoxic activity was mediated by apoptosis as it was confirmed by externalization of phosphatidylserine, DNA frgmentation, dyskinetoplastidy, cell cycle arrest, loss of mitochondrial membrane potential and ROS generation in promastigotes. The same authors reported similar results when testing eugenol-rich oil of *Syzygium aromaticum* (L.) Merr. & L.M.Perry against promastigotes and intracellular amastigotes [145]. In both studies, no adverse cytotoxic effects against murine macrophages were observed. Another investigation also assayed the antiparasitic activity of *P. betle* landrace Bangla Mahoba essential oils differing in their content of eugenol against visceral Leishmaniasis [146]. The tested essential oils induced apoptosis in promastigotes and intracellular amastigotes of *L. donovani* associated to generation of ROS targeting the mitochondria without any cytotoxicity towards macrophages. *Thymus capitellatus* Hoffmans. & Link essential oil also reported anti-parasitic activity on *Leishmania* species [147]. Transmission electron microscopy evidenced aberrant-shaped cells, mitochondrial swelling and autophagosomal structures in treated promastigote cells. Moreover, externalization of phosphatidylserine, loss of mitochondrial membrane potential, and cell-cycle arrest were also reported.

## 4. Essential Oil in Traditional Medicines: Case Studies

Essential oils have been used for over 5000 years for a variety of different purposes, including personal care (i.e., perfumes and cosmetics), foods, home care, repellents for humans and animals (livestock and domestic animals), and health-promoting agents for the treatment of various diseases. Despite differences in chemical composition of essential oils obtained from different plants with diverse preparation methods, their main constituents belong to the same chemical classes, such as mono- and sesquiterpenes (Figure 2), aldehydes, ketones, ethers and esters, alcohols and hydrocarbons. The presence of these compounds determines both chemico-physical properties (i.e., liquid at room temperature, soluble in organic solvents and insoluble in water) and biological properties such as antibacterial, antifungal, antioxidant, spasmolytic, carminative, hepatoprotective, and analgesic activities.

Many examples of essential oils used in traditional medicine can be cited. The genus *Eucalyptus* (Myrtaceae), native to Australia, is one of the most widely distributed planted genera worldwide. It includes approximately 900 species, of which about 300 species contain volatile oil and under 20 species have traditionally been used as herbal remedies. Antiseptic, antimicrobial, anti-inflammatory and antipyretic properties have been ascribed to the essential oils of certain *Eucalyptus* species, such as *Eucalyptus globulus* Labill., *Eucalyptus citriodora* Hook., *Eucalyptus gunnii* Hook. f., and *Eucalyptus camaldulensis* Dehnh. The essential oils extracted from these species are used in traditional medicine to cure various human ailments, such as diarrhea and chronic dysentery, infections of the upper respiratory tract (flu, cold, sinus congestion and fever), and toothache and oral infection [148]. Moreover, in Traditional Aboriginal Australian Medicines, *Eucalyptus* essential oil has been used as an antiseptic wash to sterilize sores, cuts, and skin infections [149].

*Mentha* genera, which belongs to Lamiaceae botanic family, include species (*Mentha piperita* L. and *Mentha spicata* L.) which grow worldwide and are cultivated on a large scale for their flavoring properties used for food and cosmetic products. Mint essential oil, obtained from mint leaves, was used for its healthy properties by many cultures in ancient times, including the Assyrians, the Babylonians, the Persians, the Carthaginians, and the Greeks. The main medicinal properties these traditions ascribed to mint essential oil include carminative properties, anti-inflammatory, analgesic, and antispasmodic activities, and diaphoretic, diuretic, and emmenagogue effects. In addition, the essential oils of some species of mint have often found external use for their astringent, rubefacient, antiseptic, and antimicrobial properties as well as the treatment of neuralgia, myalgia, headaches and migraines [150]. Clove essential oil, extracted from buds of *Syzygium aromaticum* (L.) Merr. & L. M. Perry (Myrtaceae), has found various medicinal purposes in Chinese Medicine. It has been used for mouth, skin, and genitourinary health for its antimicrobial properties. In fact, clove oil is active against oral bacteria associated with dental caries and periodontal disease, *Staphylococcus aureus*, involved in the pathogenies of acne, and *Candida albicans*, which is the most common infectious agent, responsible for mucocutaneous candidiasis (i.e., oropharyngeal and genitourinary candidiasis, which include vulvovaginal candidiasis in women, and balanitis and balanoposthitis in men). Moreover, undiluted clove essential oil has been traditionally used on gums for its anti-inflammatory properties, aiding in the treatment of toothache and various dental disorders. In addition, clove oil is used for the treatment of digestive disorders and gastrointestinal diseases in Ayurvedic medicine, acting as a carminative [151,152].

Another example of an essential oil widely used in several traditional medicines is nutmeg essential oil, which is extracted through steam distillation of ground seeds of *Myristica fragrans* Houtt. (Myristicaceae). Arabian Traditional Medicine used this essential oil as a remedy for digestive disorders (stomachache) for its analgesic and carminative properties, and its ability to stimulate the secretion of digestive enzymes and gastric juices. Moreover, it has been used for its aphrodisiac effects. Traditional Chinese Medicine used nutmeg essential oil for its analgesic properties, to treat dysmenorrhea, painful menstruation, abdominal pain and liver problems. Ayurvedic uses of nutmeg essential oil include memory improvement and tonic effects on nervous, respiratory, and circulatory functions [153,154,155].

### 4.1. Traditional Iranian Medicine

The medical use of oils in Traditional Persian Medicine (TPM) dates back prior to 637 AD [156]. However, there is limited information concerning medical practices of that period. One of the most important documents, Bondahesh, a Sassanid Pahlavi manuscript, classified all plant species into 11 groups, including oily herbs that were identified through their oily seeds [157]. Examples of medicinal herbs mentioned in the Bondahesh include olive oil (*Olea europaea* L., Oleaceae), castor oil (*Ricinus communis* L., Euphorbiaceae) and hemp (*Cannabis sativa* L., Cannabaceae) [157,158].

Generally, pharmaceutical dosages of medicinal oils were prepared by two primary methods: direct extraction from the herbs via compression of oil-bearing components or distillation of aromatic plant parts, or indirect, which involved the extraction of plants to prepare vegetable oils [159]. In the latter method, soft, fragrant aerial parts such as flowers, leaves or fleshy fruits were soaked in traditionally prepared almond, sesame and olive oils, among others, and exposed to the sun or an artificial heat source for several days, while replacing the spent parts with fresh ones until to reach a particular color and aroma (maceration in heated oil) [160,161].

Medicinal oils (Adhaan) have been used in TPM for thousands of years to treat various ailments. Currently, many of these formulations are used as ethnomedical preparations by traditional practitioners in Iran [159]. The effleurage method or oil infusion, which is a simple mean of extracting oil-soluble ingredients from plants, can be used in the traditional preparation method of herbal oils. Of the 31 plant families noted in the historical documents, most medicinal plant species used to prepare oils belonged to families of Apiaceae (six species) and Asteraceae (five species). Also, most of the oils were derived from leaves, fruits and flowers. Distillation was the most common direct extraction method of medicinal oils, whereas the most common indirect methods were maceration in heated oil and boiling and evaporating.

Several traditional oils were prepared by a boiling and evaporation method [162], in which chemical constituents in the aqueous phase become trapped in the oil phase following evaporation. However, large amounts of heat-sensitive components extracted in the aqueous phase may decompose by overheating. As this method is not well accepted in current pharmaceutic methodology, we found no credible evidence indicating the use of this form of preparation in contemporary science. Anyway, for most representative herbs, efficacy was confirmed via current pharmacological methods. Importantly, current extraction methods typically employ ethanol, methanol or aqueous extractions, in which hydrophilic ingredients are initially extracted in water and the solution is then boiled. Although aqueous extraction procedures for plant oils continue to be widely used, they are quite different from the historical methods [163]. For some of the oils cited in the historic literature that were prepared by oil infusion (maceration in heated oil), we considered clinical research which evaluated aqueous fractions. However, aqueous fractions certainly have no constituent comparable to those extracted by the oil infusion process. In contrast, organic extractions with nonpolar solvents likely extract similar components.

Medicinal oils have been traditionally used via topical, oral and even nasal routes to target particular areas of the body to combat specific ailments. Oils for gastrointestinal, respiratory, urinary and reproductive interventions were administered orally, while the nasal route was considered for disorders affecting the central nervous system. Topical forms were most often applied for nervous, musculoskeletal and integumentary afflictions. Some traditional applications reported in the Persian literature correspond to current applications. Out of the cited medicinal oils, 31 herbs comprised the main components and reportedly showed pharmacological effects in medieval Persian reports, in which analgesic and anti-inflammatory activities were the most relevant pharmacological properties. Most of the effects have been confirmed by recent in vitro or in vivo studies. Only one study on humans relevant to the traditional report was found [164]; hence, there is a lack of related human studies not only for oils, but also for other herbs and their dosages.

Notably, only a few pharmacological effects of essential oils noted in the ancient literature can be directly matched to current reports, such as clove and damask rose oils as analgesic agents [165,166], cinnamon oil for its carminative and antimicrobial properties [53,167] and the sedative effect of bitter orange [168]. There are, however, several modern reports documenting the effects of traditional medicinal oils, such as the anti-inflammatory and neuroprotective activities of terebinth oil [169,170], antiepileptogenic and neuroprotective properties of black cumin oil [171] and the xanthine oxidase inhibitory effect of almond oil polyphenols [172], which can be considered as evidence of these medicinal oils effectiveness.

#### 4.1.1. Preclinical Studies

Over the past decade, a great interest has been focused by the Iranian medicine on the use of plant oils in the treatment of epilepsy. Although no clinical study is available as regards this application, several in vivo experiments would corroborate a potential anticonvulsant activity of many traditional Iranian essential oils.

Pourgholami et al. [173] have evaluated the effect of an essential oil of *Eugenia caryophyllata* Thunb (Myrtaceae), an antiepileptic remedy in Iranian traditional medicine, against seizures induced by maximal electroshock (MES) or pentylenetetrazole (PTZ) in male mice. The essential oil obtained by distillation of dried buds was administered to male mice (21–28 g) at increasing concentrations (2.5–100 µL/mL) in a volume not higher than 10 mL/kg of body weight, using sesame oil as control. Then, seizures were induced by employing each of the following methods: PTZ (1.0%) at the dose of 85 mg/kg (minimal dose needed to induce convulsions) injected i.p. to induce clonic-tonic convulsions in animals; electroconvulsive shock (150 V, 25 Ω, 50 pulses/s, 0.2 s duration) to induce tonic hind limb extension. Essential oil at the dose of 0.050 mL/kg and higher, significantly suppressed tonic electroshock-induced convulsions and mortality only at a 10 min injection–stimulation interval, while increased the dose of i.v. infused PTZ required to produce general clonus in unrestrained mice (peak effect, 10 min), in a dose-dependent manner. Essential oil up to dose of 0.1 mL/kg had no anticonvulsant activity against PTZ-induced tonic–clonic seizures. By adopting a similar experimental protocol, the same authors have demonstrated analogous effects of EO from *Pimpinella anisum* L. (Umbelliferae) fruits [174], and Sayyah et al. [175] have described the same activity by testing the essential oil from *Laurus nobilis* L. (Lauraceae) leaves. In all of these studies, the anticonvulsant activity observed may be related mainly to eugenol, estragole, and carvacrol, present in the plants [176], and previously described [177].

More recent studies have highlighted much attention on the antimicrobial properties, especially antifungal activity, of different traditional Iranian essential oils.

Firuzi et al. [178] have tested essential oils from aerial parts of *Heracleum persicum* Desf. ex Fisch., C. A. Mey. & Avé-Lall. (Umbelliferae) against six bacterial (*Bacillus subtilis*, *Escherichia coli*, *Klebsiella pneumonia*, *Pseudomonas aeruginosa*, *Salmonella typh*i, and *Staphylococcous aureus*), and two fungal strains (*Aspergillus niger* and *Candida albicans*). None of the essential oils showed significant activity against Gram negative, Gram positive or fungal microorganisms.

Sharifi-Rad et al. [179] investigated the antimicrobial activity of *Pulicaria vulgaris* Gaertn essential oil against Gram-positive bacteria (*Bacillus cereus* and *Staphylococcus aureus*), Gram-negative bacteria (*Escherichia coli* and *Pseudomonas aeruginosa*) and fungi (*Aspergillus niger* and *Candida albicans*). Results of antibacterial test of *P. vulgaris* essential oil showed that all assayed concentrations significantly (*p* < 0.05) inhibited the growth of *B. cereus*, *S. aureus*, *E. coli*, and *P. aeruginosa*. Minimum inhibitory concentrations (MICs) for *B. cereus*, *S. aureus*, *E. coli*, *P. aeruginosa* were 17.5, 25.2, 19.4, and 33.2 μg/mL, respectively. Antifungal screening of the essential oil of *P. vulgaris* showed that the oil significantly inhibited the growth of *A. niger* and *C. albicans* (MIC = 15.5 and 9.9 μg/mL, respectively).

Sharifi-Rad et al. [180] evaluated EOs from fresh leaves of *Xanthium strumarium* L. against Gram-positive and Gram-negative bacteria and fungi The antibacterial and antifungal screening of the essential oil showed that all assayed concentrations significantly inhibited the growth of *Staphylococcus aureus*, *Bacillus subtilis*, *Klebsiella pneumoniae*, *Pseudomonas aeruginosa*, *Candida albicans* and *Aspergillus niger* (MIC = 0.5 ± 0.1, 1.3 ± 0.0, 4.8 ± 0.0, 20.5 ± 0.3, 55.2 ± 0.0, and 34.3 ± 0.0 μg/mL, respectively).

Sharifi-Rad et al. [181] have reported slight antibacterial and antifungal activities of Satureja intermedia C. A. Mey essential oil against *Streptococcus mutants*, *S. salivarius*, *Enterococcus faecalis*, *Staphylococcus aureus*, *Candida albicans* and *C. glabrata*.

The same authors [182] investigated the antibacterial and antifungal activities of essential oils from aerial parts of *Lallemantia royleana* Benth. Antibacterial screening of *L. royleana* essential oil showed that all assayed concentrations significantly (*p* < 0.05) inhibited the growth of *Staphylococcus aureus*, *Bacillus subtilis*, *Klebsiella pneumoniae* and *Pseudomonas aeruginosa*. MICs for *S. aureus*, *B. subtilis* and *K. pneumoniae* were 5.6, 4.8 and 3.5 μg/mL, respectively; *L. royleana* oil was inactive against *P. aeruginosa* in this study. Also antifungal screening showed that essential oil of *L. royleana* significantly inhibited the growth of *Candida albicans* and *Aspergillus niger* (MIC = 3.1 and 2.5 µg/mL, respectively).

Abdollahi et al. [183] have demonstrated significant effects of *Zataria multiflora* Boiss. EOs, an Iranian thyme-like medicinal plant, on the growth and sporulation of *Aspergillus niger* both in vitro and on lime fruits. In vitro antifungal assays were carried out by measuring direct fungal inhibition of essential oil for both mycelial growth and sporulation, at different concentrations (500–2000 µL in 0.5 mL Tween 80), while fruits wounded and inoculated with 0.1 mL of spore suspension (10^5^ spores/mL) of *A. niger* were dipped into 200, 400 and 600 µL/L concentrations of essential oil. EC_50_ of essential oil for mycelial growth in direct method was 819.3 µg/L, which indicates a 50% reduction in fungal growth at this concentration. Mycelial growth of fungal cultures with essential oil treatment was significantly reduced by up to 8 days post-incubation. Furthermore, a significant positive correlation was observed between percent mycelial growth inhibition and essential oil content. The fungal growth was completely prevented at 2000 mg/L. Essential oil of *Z. multiflora* also significantly inhibited sporulation of *A. niger*. Fungal sporulation decreased steadily by increasing essential oil concentrations, and at concentration ≥1500 mg/L, no sporulation occurred. The results showed that the storage life of fruits treated with 600 mg/L essential oil in the cold room was increased by 50 days, and compared with control, fruit rot started with a 10-day delay. Only 15% of the fruits treated with 600 mg/L essential oil and stored in the cold room were rotten at 50 days of storage. Other interesting findings due to the essential oil treatment were the increase of storage and shelf life of lime fruits and no changes in the appearance and quality of the fruits. Among other studies reporting similar appreciable results concerning the effects of essential oils from typical Iranian plants on fungal strains of agricultural and food interest [184,185], Shokri et al. [186] have successfully demonstrated the anti-*Candida zeylanoides* activity of the essential oils of five Iranian medicinal plants, namely *Trachyspermum copticum* (L.) Link., *Z. multiflora*, *Nigella sativa* L., *Ziziphora clinopodioides* Lam. and *Heracleum persicum* Desf. ex Fisch., C.A.Mey. & Avé-Lall. Clinical strains of *C. zeylanoides*, obtained from different parts of subjects’ genitalia, were incubated with essential oils isolated by water distillation from the aerial parts and seeds, and tested in comparison with reference standards, such as fluconazole, ketoconazole and nystatin. The essential oils of *T. copticum* and *Z. multiflora* showed a significant (*p* < 0.05) antifungal activities against *C. zeylanoides*, while the essential oils of the remaining plants showed a moderate to weak inhibition. In a previous study conducted by Naeini et al. [187], the essential oil of *Z. multiflora* showed a significant (*p* < 0.05) antifungal activity against *C. albicans*, while the essential oils of *Z. clinopodioides*, *N. sativa* and *H. persicum* exhibited a moderate to weak inhibition. Although essential oil concentrations were high when compared with those of the antifungal drugs, these results are of interest as dealing with complex mixtures and not a pure product. Shokri et al. [186] have indicated thymol (63.4%) and r-cymene (19%) as main compounds from *T. copticum* oil. *Z. multiflora* main components were carvacrol (61%) and thymol (25%). Trans-anthol (39%) and r-cymene (17%) were identified from *N. sativa* oil. The GC/MS analysis of *Z. clinopodioides* and *H. persicum* essential oils showed pulegone (37%) and aperitone (19.6%), and hexyl butyrate (30.2%) and octyl acetate (12.8%), respectively, as main constituents.

#### 4.1.2. Clinical Studies

Very recent clinical trials have highlighted specific positive effects of traditional Iranian essential oils on both the physiological conditions and psychological sphere in human subjects. Interestingly, the most significant in human studies have focused the attention on the healthy potential of lavender (*Lavandula angustifolia* Mill.) essential oil, whose peculiar chemical composition has been indicated as responsible for many of the biological effects observed.

Sheikhan et al. [188] have revealed a good capacity of lavender essential oil to relieve episiotomy/perineal pain in primiparous Iranian women. Essential oil, extracted from fresh flowers and inflorescences, was employed in comparison with betadine used routinely to improve the healing of episiotomy wounds. Two groups of 60 primiparous mothers, with single tone vaginal deliveries with episiotomy, were randomly allocated to either the experimental or control group. The use of lavender oil resulted in statistically significant differences 5 days after episiotomy, compared with betadine use by the control group. Among the different chemical constituents of lavender essential oil, linalyl acetate and linalool are regarded as the main responsible for sedative and local anesthetic effects [189,190]. Linalool can increase the local blood circulation and also reduces muscle tone, thus providing analgesic and sedative properties [191]. This study suggests that the application of lavender essential oil on the perineum following episiotomy may be an effective form of pain relief and enhance the wound healing process.

Lavender essential oil has been also tested for its potential effects on the symptoms of primary dysmenorrhea and the amount of menstrual bleeding through inhalation [192]. A randomized clinical trial included 96 female subjects suffering from level two or three primary dysmenorrhea according to the Andersch and Milsom’s verbal multi-dimensional scoring system [193]. The intervention group used lavender aroma diluted in sesame oil in a 2:1 ratio, and the placebo group used sesame oil only. The participants were asked to strew 3 drops of the treatment on their palms, rub them together, keep their hands at the distance of 7–10 cm from their nose and inhale for 5 min. The treatments were administered to the subjects for 1 h after experiencing dysmenorrhea. They were asked to use the treatments every 6 h for the first three days of menstruation. During the two consecutive menstrual cycles, one of the two treatments (placebo or lavender) was administered to the subjects. Results showed that lavender essential oil was highly effective (*p* < 0.001) in reducing the score of symptoms (mood change, faint, hot flushing, nasal congestion, abdominal pain, backache, tiredness, nausea, headache). Moreover, the estimated odds of moderate and heavy bleedings in the lavender group were 1.4, 0.7 and 0.3 times the estimated odds of moderate and heavy bleedings in the control group in the first, second and third day of menstruation, respectively.

A further investigation on the potential bioactivity of lavender essential oil through inhalation has consisted in assessing its effects on stress and vital signs in patients undergoing coronary artery bypass graft surgery (CABG) [194]. A single-blinded, randomized, controlled trial was carried out with 60 patients following CABG. The patients inhaled two drops of 2% lavender oil in alcohol from an absorbable sticky patch inside an oxygen mask for 20 min on the second and third days after surgery. The patients in the control group inhaled two drops of distilled water as a placebo via an oxygen mask for the same period as the aromatherapy group. On the second and third days after surgery, the mental stress levels in the aromatherapy and control groups was measured before and 60 min after aromatherapy using the DASS-21 questionnaire [195]. The vital signs, i.e., the heart rate, respiratory rate, and systolic and diastolic blood pressure, were measured before and at 5, 30, and 60 min after aromatherapy. The mean mental stress score decreased significantly immediately after surgery in both groups, but there were no significant difference between the aromatherapy and control groups before and after intervention on the second and third days after surgery. On the second day, the heart rate was faster in the aromatherapy group and faster on the third day in the control group. The respiratory rate was faster in the aromatherapy group on both the second and third days. On the second day, the systolic blood pressure was higher in the control group and on the third day in the aromatherapy group, while the diastolic blood pressure was higher in the aromatherapy group on both the second and third days. However, there was no significant difference between the vital signs in the aromatherapy and control groups on the second and third days after surgery, except for the systolic blood pressure on the third day after 5 and 30 min, and the diastolic blood pressure on the third day after 5 min.

Another interesting in human study regarding the influence of lavender essential oil on the psychological sphere concerns its effects on preoperative anxiety in patients undergoing diagnostic curettage [196]. In this clinical trial, 100 patients were divided into two groups randomly assigned to the intervention group (*n* = 50) and the control (*n* = 50). Spielberger’s state anxiety inventory with 20 items and the VAS questionnaire (visual analogue scale) were filled out by the two groups before and after aromatherapy. Lavender patients inhaled essential oil for 60 s and, to be stable, the patient’s nose was shortly massaged with the extract, whereas the control group was treated with 0.1% lemon using the same procedure. This study revealed that the apparent anxiety level in patients who inhaled lavender essential oil was noticeably lower than before intervention results and also compared with control group, especially at the levels of severe and highly severe anxiety. The mechanism of action of lavender essential oil has not been completely investigated, but, according to various studies, its psychological effects may derive by affecting limbic system, especially amygdala and hippocampus, where there could be activities similar to benzodiazepines and gamma aminobutyric acid [197]. According to Re et al. [198], linalool would inhibit acetylcholine release, changing the function of ion channels at neuromuscular joints, while linalyl acetate would play a narcotic function, acting as a sedative.

### 4.2. Ayurvedic Medicine

Ayurveda, the classical Hindu traditional medicine, has been in practice in many countries for centuries. Ayurvedic medicine can be defined as the administration of oral remedies according to the principles of Ayurveda. One of the key concept of Ayurveda is Svastha, or a healthy living being [199]. This is a being that possesses the equilibrium of doshas (the triad of physiological functional elements), with proper functioning of dhatus (body tissues), agni (digestive system and enzymes of the metabolic pathways), mala (excretory system and by-products of metabolism), and presence of indriya (sensory functions), manah (mental faculty) and atma (self) [199]. Ayurveda basically focuses on the whole being and not with the disease alone; hence, it is popularly referred to as the ‘mind-body medicine’ [199]. Ayurvedic medicine makes use of medications that primarily consist of herbs, metals, minerals, or other materials [200].

Several scientific studies are on-going to give scientific validation for the use of the components of Ayurvedic medications; this is aimed to channel regulations and acceptance for the clinical practice. Aromatic plants which are rich in essential oils represent one of the major ingredients of Ayurvedic medications. The use of essential oils in Ayurvedic traditional and complementary medicine is gaining more popularity over the years. This is due to their well-studied biological effects which include antimicrobial [201,202], phsycoactive [202,203], and antidiabetic [204] activities, among others. An overview of the preclinical and clinical studies of essential oils from some common plants used in Ayurvedic medications is presented in the following sections.

#### 4.2.1. Preclinical Studies

##### Diabetes and Related Complications

Diabetes mellitus (DM) is characterized by hyperglycemia (high blood glucose levels) due to the inability of the pancreas to produce enough insulin or of the cells to respond to the insulin that is produced [205]. Type 2 DM (T2DM) is the most common form of diabetes [206] which features insulin resistance and/or relatively reduced insulin secretion [205], leading to hyperglycemia and, ultimately, malfunctioning of the pancreatic β-cells [205]. Sustained hyperglycemia leads to high generation of free radicals such as reactive oxygen species (ROS) and impairment of endogenous antioxidants [207]. The resulting oxidative stress has been implicated in the impairment of the pancreatic β-cells and associated diabetes complications [205]. The treatment of diabetes and associated complications such as hypertension is being constantly researched; however, sustainable therapies for these conditions are still a long way off [205]. The major therapeutic management for diabetes still remains the systematic reduction of glycemia via inhibition of α-amylase and α-glucosidase which are the major carbohydrate metabolizing enzymes. Hence, major drugs used in the management of diabetes are inhibitors of these enzymes, such as acarbose, which present a number of side effects [205]. Consequently, research efforts are geared towards the use of natural products, especially from plant foods or extracts for the management of diabetes and other related health complications. In view of these, plant essential oils have been well explored for these properties.

In a study conducted by Ademiluyi et al. [204], the inhibitory activity of the essential oil from sweet basil (*Ocimum basilicum* L.) leaves was investigated in vitro on α-amylase, α-glucosidase and angiotensin-I-converting enzymes (ACE). In addition, these authors also investigated the antioxidant properties of the essential oil via inhibition of Fe^2+^ and sodium nitroprusside (SNP)-induced lipid peroxidation in rats’ pancreas and heart tissue homogenates. The phytoconstituents of the oil were analyzed using gas chromatography (GC). Sweet basil essential oil inhibited the activities of α-amylase (IC_50_ = 3.21 mg/mL), α-glucosidase (IC_50_ = 3.06 mg/mL) and ACE (IC_50_ = 0.89 mg/mL), as well as both Fe^2+^ and SNP-induced lipid peroxidation in tissue homogenate. The bioactivities were largely correlated with the main constituents of *O. basilicum* essential oil, including limonene (47.40%), borneol (8.66%), geranial (6.93%), neral (5.71%), myrcene (4.68%), β-caryophyllene (4.68%), α-terpineol (4.60%), 1,8-cineole (4.17%), linalool (3.53%), β-elemene (3.05%), germacrene D (2.68%), and terpinen-4-ol (2.21%). Hence, this plant species widely known in Ayurvedic practices could offer protection against diabetes and its complications via its enzyme inhibitory and antioxidant properties.

In another study, Oboh et al. [208] investigated the in vitro antidiabetic and antioxidant potentials of essential oil from clove bud (*Eugenia aromatic* O.Berg). Clove bud is a spice of vast culinary and medicinal values. Its bioactive properties have been largely ascribed to its constituent phytochemicals such as the terpenoids, monoterpenes and sesquiterpenes. In their study, Oboh et al. [208] observed that the hydrodistilled essential oil from clove bud inhibited the activities of α-amylase and α-glucosidase, besides exhibiting antioxidant properties. GC-MS characterization of the oil revealed the presence of α- and β-pinene, neral, linalool and γ-terpene among others. Oboh et al. [209] also studied the antidiabetic and antihypertensive properties of essential oil of another spice, black pepper (*Piper guineense* Schumach. & Thonn.) seeds, in an in vitro system by assessing their α-amylase, α-glucosidase (key enzymes linked to type-2 diabetes), and angiotensin-I converting enzyme (ACE) (key enzyme linked to hypertension) inhibitory activity. According to them, the essential oil inhibited the three enzymes concentration dependently, while also exhibiting significant antioxidant activity. These bioactivities were attributed to the individual compounds in the essential oil which GC characterization revealed to include α-pinene, β-pinene, *cis*-ocimene, myrcene, allo-ocimene, and 1,8-cineole. Therefore, they concluded that the phenolic content, antioxidant activity, and inhibition of α-amylase, α-glucosidase, and angiotensin-I converting enzyme activities by the essential oil extract of black pepper could be part of the mechanism by which it could be used to manage and/or prevent type-2 diabetes and hypertension.

##### Cancer and Radiotherapy

According to traditional Lebanese medicine, *Salvia* species such as *Salvia aurea* L., *Salvia judaica* Boiss. and *Salvia viscosa* Jacq. are popular among many local indigenes for the therapeutic properties of their essential oils [210]. These *Salvia* species are often used as Ajurvedic components in many multiherb products commonly patronized in Lebanon and surrounding countries for the treatment of cancer and other diseases [211]; nevertheless, there is dearth of scientific information supporting their use. Russo et al. [210] investigated the qualitative and quantitative compositions of the essential oils of these *Salvia* species, as well as their biological activity against human melanoma cells and the anticancer mechanisms involved, as cell membrane integrity, genomic DNA fragmentation and caspase-3 activity. The authors reported that all the essential oils were able to decrease the growth of cancer cells by inducing apoptotic cell death. These activities were associated to their constituents which predominantly includes sesquiterpenes, particularly oxygenated sesquiterpenes with caryophyllene oxide.

Radiotherapy is a treatment option in oncological practices. However, the search for radioprotective agents to selectively protect non-target tissues against radiation injury is a pivotal and current topic [212]; this will offer higher protection for the healthy tissues during cancer treatments, thus improving disease management [212]. Unfortunately, the availability of an ideal and safe radioprotector agent has been greatly limited, while most synthetic compounds such as aminothiol, *S*-2-(3-aminopropyl-amino) ethyl phosphorothioic acid (WR-2721), amifostine, ethiophos, and gammaphos are toxic especially at their optimal concentrations which greatly limits the therapeutic benefits they offer [213,214,215]. Interestingly, phytochemicals are being explored for their radioprotective properties.

*Ocimum sanctum* L. popularly known as the Holy Basil is an aromatic herb that belongs to the family Lamiaceae [212]. The plant originated from India, where it has the local name Tulsi (meaning ‘the matchless one’), but has also been found growing in tropical Asia, some areas in north and eastern Africa, as well as some regions in China [212]. Tulsi is mostly cultivated for its religious and medicinal uses which are greatly derived from its essential oil components [212]. This plant is an highly referred drug in Ayurvedic medicine, where it is often used either alone or in combination with some other plants in the treatment of disorders such as different forms of skin diseases, rheumatism and arthritis and heart diseases [212,216,217].

Phytochemical profiling of the leaf of Tulsi has revealed that it is rich in volatile oil with eugenol, methyl eugenol, carvacrol and caryophyllene as main constituents [213,216,217]. In addition, phenolic compounds such as cirsilineol, circimaritin, isothymusin, apigenin and rosmarinic acid have been characterized from fresh leaves and stem of Tulsi [212]. The medicinal properties of Tulsi have been largely ascribed to these phytochemicals and especially the volatile oil components. The radioprotective properties of Tulsi extract have been largely investigated. It has been shown that the combination of Tulsi with 100–400 mg/kg amifostine (a registered radioprotective drug with deleterious side effects) significantly reduced the cell damage of the mouse bone marrow following whole-body gamma-irradiation exposure (4.5 Gy) for 14 days [218]. In addition, combining Tulsi with amifostine reduced the toxic side effects of this drug at high dose, thus suggesting a better approach at radioprotection [218]. The mechanism of action of Tulsi as radioprotectant agent is due to its antioxidant properties via free radicals scavenging activity and possible stimulation of endogenous antioxidant enzymes [212].

##### Neurodegenerative Diseases

Neurodegenerative diseases represent a group of chronic disorders characterized by progressive and selective decline in neuronal and cognitive functions, found in about 5% of reported cases of brain diseases [219]. The unique pattern by which each neurodegenerative disease causes progressive neuronal damage and their ability to produce disease-specific cellular biomarkers have been of importance in their classification [220]. Alzheimer’s disease (AD) is a neurodegenerative disorder characterized by decline in acetylcholine (ACh) neurotransmitter, deposition of senile plaques, neurofibrillary tangles and progressive loss of cognitive function [219,220]. Another typical neurodegenerative disorder is Parkinson’s disease (PD) characterized by generation of Lewy bodies and depletion of dopamine neurotransmitter, while cellular inclusions and swollen motor axons are found in amyotrophic lateral sclerosis [221]. Huntington’s disease (HD) features loss of neurons containing γ-aminobutyric acid [222]. Although these diseases can affect all individuals, they are more common among the aged people, showing a higher incidence in aging [220]. The possible contribution of environmental factors to the pathogenesis of some neurodegenerative diseases is gaining more attention [223]. For example, environmental pollutants like pesticides and heavy metals, plant derived toxins, and some drugs could possibly contribute to the onset of PD [224].

Several studies have reported the potential neuroprotective properties of essential oil from a number of plants used either alone or in combination with other plants in many traditional medical practices. In one study, essential oil from clove bud (*Syzygium aromaticum* (L.) Merr. & Perry) and Ethiopian pepper (*Xylopia aethiopica* Dun. A. Rich, Annonaceae) showed neuroprotective properties in vitro by exhibiting anticholinesterase and antioxidant activities [225]. These essential oils also exhibited membrane-stabilizing properties by inhibiting quinolinic acid induced lipid peroxidation in rat brain homogenate [225]. In another study, essential oils from peels of sweet orange (*Citrus sinensis* (L.) Osbeck) [226] and lemon (*Citrus limon* (L.) Osbeck) [227] were investigated for their in vitro antioxidant and membrane stabilizing properties, and inhibitory activity of acetylcholinesterase (AChE) and butyrylcholinesterase (BChE) enzymes. The authors observed that, based on these biological activities, essential oils could be used in the treatment of neurodegenerative disorders, especially Alzheimer’s disease.

#### 4.2.2. Clinical Studies

Despite the huge amount of preclinical studies on the pharmacological properties of essential oils, there exits a paucity of in human clinical trials. In a systematic review of randomized clinical trials by Perry et al. [228], lavender essential oil was investigated as an anxiolytic drug. In particular, different methods of administering lavender were examined. Eight trials were based on aromatherapy [229,230,231,232,233,234,235]; two trials were based on massage [236,237]; one study used an oil-dripping technique [238]; one trial was based on bathing in lavender oil [239]; while, in three studies, lavender capsules were orally administered [240,241,242]. Authors concluded that oral route of administration was the most promising in terms of anxiolytic effects on the subjects, with hope of more of such trials to confirm lavander efficacy, with long term follow up.

### 4.3. Traditional Korean Medicine

Traditional Korean Medicine (TKM) is one of the traditional East Asian medical systems that, for more than 2000 years, has been used to treat several diseases, including cancer [243].

A holistic approach including the concepts of the body-mind-spirit network, the balancing theory of Yin-Yang and Five Phases, is the basis of TKM, which is characterized by an equal emphasis on the individual’s differences as well as symptom pattern differentiation [244,245]. Regarding cancer treatment, TKM puts emphasis on the modulation and improvement of the whole body rather than just killing cancer cells. In recent years, TKM-based medicinal plants gained renewed interest in cancer prevention and treatment. Some medicinal plants that have been used to treat Jeok-chi (closely linked to abdominal tumours accumulated over a long period) or Ong-juh (disease which refers to all acute and chronic inflammation, including abscesses and carbuncles, related to cancer), have been comprehensively studied for their anticancer effects.

#### Preclinical Studies

The essential oils of some medicinal plants used in TKM have been investigated for their potential anticancer activity (Table 1). Among these, plants from Amaryllidaceae, Burseraceae, Compositae, Solanaceae and Zingiberaceae showed interesting results.

*Allium sativum* L. (Amaryllidaceae) has shown an anti-proliferative effect on human breast, colon, lung, skin and liver tumors [246]. In particular, *A. sativum* oil increased glutathione (GSH) peroxidase activity in isolated epidermal cells incubated in the presence or absence of a tumor promoter 12-*O*-tetradecanoylphorbol-13-acetate (TPA), and inhibited the sharp decline in the intracellular ratio of reduced (GSH)/oxidized (GSSG) glutathione caused by TPA. The stimulatory effects of this oil on epidermal GSH peroxidase activity are concentration-dependent and long lasting, thus abolishing the prolonged inhibitory effect of TPA on this enzyme. In addition, garlic oil (5 µg/mL) inhibited by about 50% TPA-induced ornithine decarboxylase (ODC, l-ornithine carboxylyase, EC 4.1.1.17) activity in the same epidermal cell system. This concentration of garlic oil remarkably increased GSH peroxidase activity and inhibited ODC induction in the presence of various non-phorbol ester tumor promoters. According to these findings, it was postulated that the inhibitory effects of garlic oil on skin tumor promotion might result from the enhancement of the GSH-dependent antioxidant protective system of the epidermal cells [264].

*Boswellia* species (Burseraceae), which are trees native to Ethiopia, Somalia, India, and Arabic peninsula, produce a gum resin that is known as frankincense. This gum resin has long been used in Ayurvedic and traditional Chinese medicine to treat a variety of health aspects [89]. The main bioactive constituents of resin are boswellic acids. These acids have also been shown to possess potential chemopreventive effects, e.g., they inhibited the growth of brain tumor [265] and meningioma cells [266], as well as they induced apoptosis in human leukemia cells [267].

Gas chromatography-mass spectrometry (GC-MS) analysis of the *Boswellia serrata* Roxb. ex Colebr. volatile oil revealed the presence of sabinene (19.11%), terpinen-4-ol (14.64%) and terpinyl acetate (13.01%) as major constituents. Treatment of HepG2 cells with volatile oil showed a significant and dose-dependent reduction of cell viability with IC_50_ value of 5.5 g/mL after 48 h. Likewise, the treatment of HCT116 cells resulted in a dose-dependent decrease in cell viability with IC_50_ value of 6.2 μg/mL after 48 h [247].

*Artemisia capillaris* Thunb. (Compositae) has been used in traditional Korean medicine for their interesting biological activities including antioxidant, anti-inflammatory, hepatoprotective and anticancer properties [95,268]. Cha et al. [95] investigated the ability of *A. capillaris* essential oil to induce apoptosis in the human oral epidermoid carcinoma cell line KB. The oil included capillene (32.7%), α-caryophyllene (11.1%), and α-pinene (9.4%) as main constituents [269]. The essential oil reduced the viability of KB cells in a concentration- and time-dependent manner. Poly (ADP-ribose) polymerase (PARP) proteins are important markers of programmed cell death. A dose-dependent increase in the level of the PARP 85 kDa cleaved forms from the 116 kDa origin proteins after the *A. capillaris* essential oil treatment was observed. Moreover, condensation and fragmentation of nuclei, and cellular shrinkage (distinctive apoptotic features) were evidenced in the cells after treatment with the oil (at a concentration of 0.5 μL/mL for 12 h). After treating KB cells with the *A. capillaris* essential oil, the concentration-dependent activation of caspase-8 was observed. The involvement of the c-Jun *N*-terminal kinase (JNK)/Bcl-2-mediated and p38/nuclear factor-kappaB (NF-κβ) pathways as well as caspase activation in the *A. capillaris* essential oil-mediated apoptosis was also demonstrated.

*Artemisia iwayomogi* Kitam. (Compositae) is a small herbal plant that has long been used in traditional Korean medicine as chemopreventive agent [248]. The chemical profile of *A. iwayomogi* essential oil revealed the presence of camphor (19.31%), 1,8-cineole (19.25%), borneol (18.96%), camphene (4.64%), and α-caryophyllene (3.46%) as the most abundant compounds [270]. The potential chemopreventive activity of this essential oil against the human oral epidermoid carcinoma cell line KB was studied [248]. *A. iwayomogi* essential oil induced apoptosis of KB cells, mediated by mitogen-activated protein kinases (MAPKs). *A. iwayomogi* essential oil induced the cleavage of PARP and the activation of caspases. An imbalance between the mitochondrial levels of Bcl-2 and Bax was also observed. Moreover, pre-treating the cells with caspase or MAPK-specific inhibitors apparently inhibited cytotoxicity of KB cells induced by the essential oil.

Another *Artemisia* species used in TKM is *A. lavandulaefolia* Nakai. The effects of *A. lavandulaefolia* essential oil on apoptosis and necrosis of human cervix adenocarcinoma (HeLa) cell line were investigated [249]. The oil inhibited the proliferation of HeLa cells in a dose-dependent manner. After treatment with *A. lavadulaefolia* essential oil (at concentration of 100 and 200 g/mL for 24 h), HeLa cells showed cell shrinkage and nucleus chromatin condensation that are typical morphological features of undergoing apoptosis. However, at the concentration 400 g/mL, HeLa cells showed necrotic morphology changes. Furthermore, the cleavage of PARP was inactivated and the caspase-3 was activated. The major constituents of the *A. lavandulaefolia* essential oil were α-caryophyllene (16.1%), *cis*-chrysanthenol (7.0%), 1,8-cineole (5.6%), borneol (5.3%), *trans*-α-farnesene (5.1%), and camphor (4.9%) [271].

*Pinus koraiensis* Siebold & Zucc. (Pinaceae) (generally called the Korean nut pine) is an evergreen tree found in Korea, China, Japan, and eastern Russia. *P. koraiensis* essential oil contains different constituents, including limonene, camphene, 4-carene, α-pinene, and α-phellandrene [272]. The antitumor mechanism of the essential oil was investigated in vitro in human colon carcinoma (HCT116) cell line [93]. *P. koraiensis* essential oil reduced the proliferation of HCT116 cells through G1 arrest, inhibited cell migration, altered cytoskeletal structure through the reduction of basal spread and cell elongation and increased cell rounding.

The genus *Solanum* (Solanaceae) comprises around 1700 species commonly found in the temperate and tropical regions of the world. Several *Solanum* species are used in Asiatic traditional medicine. *Solanum nigrum* L. is one of the herbal ingredients in prescriptions of Traditional Chinese Medicine to treat liver, mammary, uterine cervix, gastric and other cancers [250].

*Solanum spirale* Roxb. essential oil from unripe fruits was analyzed for its chemical composition and anticancer activity. *n*-Hexadecanoic acid (56.01%), linoleic acid (9.71%), octadecanoic acid (4.41%), methyl palmitate (1.69%), tetradecanoic acid (1.55%), (*E*)-phytol (1.18%), *n*-hexanal (0.91%), methyl salicylate (0.83%), 4-hydroxy-4-methylpentan-2-one (0.81%), pentadecanoic acid (0.71%) and β-selinene (0.56%) were identified as main constituents. The oil exhibited anticancer activity against breast cancer cell line (MCF-7) and small cell lung cancer (NCI-H187) with IC_50_ values of 23.17 and 49.07 g/mL, respectively. No cytotoxic activity was found against KB cell line [251]. The essential oil of *S. spirale* leaves showed significant cytotoxicity against oral cancer cells (KB), breast cancer cells (MCF-7) and small cell lung cancer cells (NCI-H187) with IC_50_ values of 26.42, 19.69, and 24.02 μg/mL, respectively. (E)-Phytol (48.10%) was the most abundant compound followed by *n*-hexadecanoic acid (7.34%), α-selinene (3.67%), α-selinene (2.74%) [252].

Osorio et al. [253] investigated the chemical composition and antileukemic activity of essential oil from *Solanum stipulaceum* Roem. & Schult. flowers. β-Caryophyllene (25.8%), caryophyllene oxide (3.6%), γ-gurjunene (11.9%), α-gurjunene (8.2%), α-selinene (5.3%), and α-humulene (2.7%) were identified as main compounds. The essential oil showed antileukemic activity against both human acute promyelocytic leukemia (HL-60) and acute monocytic leukemia (THP-1) cells after γ-radiation (10.0 kGy), with IC_50_ values of 86.67 and 40.12 g/mL, respectively. β-Caryophyllene, one of the major constituents identified in this essential oil, has been reported as a cytotoxic agent against human cancer cell lines. This sesquiterpene inhibited the constitutive activation of PI3K/AKT/mTOR/S6K1 signaling cascade, but also caused the activation of ERK, JNK, and p38 MAPK in tumor cells. Moreover, it increased reactive oxygen species (ROS) generation from mitochondria, which is associated with the induction of apoptosis, down-regulated the expression of various downstream gene products that mediate cell proliferation (cyclin D1), survival (bcl-2, bcl-xL, survivin, IAP-1, and IAP-2), metastasis (COX-2), angiogenesis (VEGF), and increased the expression of p53 and p21 [273]. In previous studies, *trans*-caryophyllene was active against renal cell adenocarcinoma (ACHN) and amelanotic melanoma (C32) cell lines, with IC_50_ values of 21.81 and 20.10 g/mL, respectively [76,274]. Another compound identified in the oil is α-humulene that showed cytotoxic activity against hormone-dependent prostate carcinoma (LNCaP) cell line with an IC_50_ value of 11.24 g/mL. This sesquiterpene was inactive against the breast cancer (MCF-7), C32, and ACHN cell lines at the maximum concentration tested (50 g/mL).

Other *Solanum* species recently reported as cytotoxic are *Solanum erianthum* D.Don and *Solanum macranthum* Dunal. *S. erianthum* leaf volatile oil demonstrated potent inhibitory activity against human breast carcinoma (Hs 578T) and prostate carcinoma (PC-3). In particular, *S. erianthum* exhibited 98.85 and 97.94% of cell mortality against Hs 578T and PC3, respectively, while *S. macranthum* oil exhibited 2% mortality against breast cancer cells. *S. erianthum* oil was characterized by the abundance of α-terpinolene (17.8%), α-phellαndrene (17.5%), *p*-cymene (15.7%) αnd α-pinene (11.7%) in the leαves; α-humulene (23.1%), humulene epoxide II (20.0%), cαryophyllene oxide (16.5%), methyl sαlicylαte (11.8%) αnd α-cαryophyllene (10.9%) in the fruits. The leαf oil of *S. mαcrαnthum* consisted of (*E*)-phytol (29.0%), pentαdecαnαl (28.1%) αnd pentαdecαne (7.7%), while the mαjor fruit oil constituents were α-humulene (36.5%), α-caryophyllene (17.8%), ethyl palmitate (9.4%), and methyl salicylate (8.2%) [254].

*Vitex rotundifolia* L.f. (Verbenaceae) has long been used in Korean traditional medicine to treat asthma and other allergic diseases [275]. Dried fruits of *V. rotundifolia* also showed strong estrogenic activity. Hu et al. [255] identified linoleic acid (47.46%), palmitic acid (5.18%), hentriacontane (2.28%), and stearic acid (2.2%) as main compounds of oil extracted by supercritical fluid extraction. The oil strongly stimulated the proliferation of MCF-7 cells. In fact, the proliferative effects of 1 nM estradiol and 50 mg/L of essential oil were significantly inhibited by the specific estrogen receptor antagonist ICI 182,780 (100 nM). Collectively, results showed that the essential oil of *V. rotundifolia* could significantly stimulate the growth of MCF-7 cells and the proliferation stimulatory effect could be reversed by co-administration of a pure anti-estrogen (ICI 182,780).

*Curcuma longa* L. (turmeric) is a perennial from the Zingiberaceae family that is widely cultivated in the tropical regions of Asia. This genus is composed of about 70 species of rhizomatous herbs, which are distributed all over the world. Turmeric contains up to 5% essential oils. An isolate from turmeric oil has been reported to have antimutagenic activity thought to be mediated through its antioxidant activity [256]. The essential oil from *C. longa* rhizome is a complex mixture obtained by steam distillation, and twelve components were identified by GC-MS analysis: ar-turmerone (61%), curlone (11.2%), and cucumene (5.5%) were the most abundant compounds [257].

Liju et al. [258] demonstrated that *C. longa* essential oil has significant antimutagenic activity against mutagens needing metabolic activation, such as 2-acetamidoflourene, and inhibites the mutagenicity induced by tobacco extract to Salmonella TA 102 strain. DMBA and croton oil induced papilloma development in mice was found to be delayed and prevented significantly by its application. Results clearly evidenced that *C. longa* essential oil inhibited isoforms of cytochrome p450 (CYP1A1, CYP1A2, CYP2B1/2, CYP2A, CYP2B and CYP3A) enzymes in vitro, which are involved in the activation of carcinogens.

*C. longa* oil was also tested against human mouth epidermal carcinoma (KB) and murine leukemia (P388) cell lines using MTT assay. The turmeric oil showed IC_50_ values of 0.0838 and 1.0879 mg/mL for P388 and KB cell lines, respectively [276]. Structure-relationship analysis revealed that the in vitro anticancer property against three different leukemia cell lines (HL-60, K-562, and L1210) of ar-turmerone is related to α,β-unsaturated ketone [277].

*Curcuma aromatica* Salisb. oil was studied for its antioxidant and anticancer activities on esophageal carcinogenesis. Severe esophagitis was evidenced in rats after esophagoduodenal anastomosis, and morphological transformation within the esophageal epithelium was observed with intestinal metaplasia after 3 months. In treated rats by i.p. injection of *C. aromatica* oil at 100 mg/kg, every 3 days, both MnSOD enzymatic activity and protein level were similar to controls. Decreased incidences of both intestinal metaplasia and esophageal adenocarcinoma were also observed [278].

Among *Curcuma* species used in TKM, *C. zedoaria* Roxb. (known as zedoary) is used for the treatment of flatulence, dyspepsia, menstrual disorders, cough, and fever. The zedoary essential oil exhibited anti-proliferative activity on mouse melanoma cells (B16BL6) and human hepatoma cells (SMMC-7721) with IC_50_ values of 41.8 and 30 g/mL, respectively. An IC_50_ of 150 g/mL was found against human umbilical vein endothelial cells (HUVEC) cells. Moreover, the essential oil significantly suppressed the sprouting vessels of aortic ring and formation of microvessels in chick embryo chorioallantoic membrane. Additionally, solid melanoma grown in left oxter of mice was inhibited after oral intake of 100 and 200 mg/kg of the oil once a day for 28 days, and CD34 expression indicating angiogenesis in melanoma reduced significantly compared with control; melanoma metastatic nodules in lung were also inhibited, as well as MMP-2 and MMP-9 expression in serum [260].

Zedoary essential oil caused a time- and concentration-dependent inhibition of non-small cell lung carcinoma (NSCLC) cell proliferation with IC_50_ values ranging from 80 to 170 g/mL in H1299 cells. The essential oil increased the sub-G1 population and the level of annexin-V binding, and induced cleavage and activation of caspase-3, -8, and-9 and poly(ADP ribose) polymerase. It decreased the levels of Bcl-2 and Bcl-xL and determined an increase in the Bax/Bcl-2 ratio. The release of AIF (apoptosis-inducing factor), cytochrome c and endonuclease G into the cytosol, and increased levels of p53 in H1299 cells was observed. Zedoary essential oil slightly inhibited the phosphorylation of ERK1/2 and AKT/NF-κβ, and enhanced the phosphorylation of JNK1/2 and p38. The highest value of cells in apoptosis (41.6%) was observed after exposure for 72 h with characteristic hallmark of DNA fragmentations. Moreover, intraperitoneal administration of zedoary essential oil significantly suppressed the growth of H1299 cells implanted subcutaneously in BALB/c (nu/nu) nude mice. The GC-MS analysis revealed the presence of 8,9-dehydro-9-formyl-cycloisolongifolene,6-ethenyl-4,5,6,7-tetrahydro-3,6-dimethyl-5-isopropenyl-trans-benzofuran, eucalyptol, and γ-elemene as main constituents [91].

*Curcuma wenyujin* Y.H.Chen & C.Ling is used as ingredient of Ezhu (*Rhizoma curcumae*) together with *C. phaeocaulis* Valeton and *C. kwangsiensis* S. G. Lee & C. F. Liang in Asiatic traditional medicines including the Korean, Chinese and Japanese ones. This remedy is traditionally used for removing blood stasis, alleviating pain, and liver disease protection. Injection of the essential oil has been used to cure pediatric diseases such as acute upper respiratory infections, acute pneumonia or viral myocarditis [259]. Moreover, *C. wenyujin* oil has shown promising effects in the treatment of cervical, gastric, liver, and lung cancers [261,262]. The treatment of hepatoma cell line (HepG2) with the essential oil resulted in a dose-dependent anti-proliferative activity with IC_50_ of 70 g/mL. The oil induced a cell cycle arrest at S/G2. This growth inhibition was associated with cell cycle arrest, cytochrome c translocation, caspase 3 activation, poly-ADP-ribose polymerase (PARP) degradation, and loss of mitochondrial membrane potential. This process involves a mitochondria-caspase dependent apoptosis pathway [279]. Shi et al. [263] reported the chemical composition and anticancer activity against gastric cancer cells of zedoary oil. Curzerene (26.45%), eucalyptol (12.04%), curcumol (9.04%), pyridine (7.97%), germacrone (7.89%), β-elemene (7.36%) and τ-elemene (4.11%) were identified as main constituents. Zedoary oil significantly decreased the cell viability of both AGS and MGC 803 cells. In AGS cells, the oil inhibited cell proliferation in a dose- and time-dependent manner and induced cell cycle arrest at S, G2/M and G0/G1 stages. At concentrations of 30, 60 and 90 μg/mL, which resulted in significant inhibition of proliferation and cell cycle arrest, zedoary oil induced cell apoptosis. In addition, Hoechst 33342/PI double-staining confirmed the morphological features of cell apoptosis at 24 h. Moreover, the oil up-regulated the Bax/Bcl-2 ratio. One of the Ezhu’s ingredients, furanodiene showed a promising anticancer activity by inducing apoptotic pathway. In particular, Xu et al. [280] demonstrated that this terpenoid concentration-dependently inhibited cell proliferation and blocked the cell cycle progressions in G1 phase by down-regulating the protein levels of cyclin D1 and CDK6, and up-regulating those of p21 and p27 in 95-D lung cancer cells. Additionally, furanodiene down-regulated PARP, procaspase-7, survivin, and Bcl-2 levels, and up-regulated cleaved PARP. Authors also demonstrated that furanodiene enhanced the expression of LC3-II, indicating that autophagy is involved in this process. Moreover, it reduced the ability of cells to adhere to the matrigel and slightly suppressed cell migration and invasion. Furanodiene also induced apoptosis in HL60 leukemia cells; DNA fragmentation, cleavage of PARP, caspase-3, caspase-8 and caspase-9 were evidenced. The terpene activated Bid protein without any effect on Bcl-2, Bax and Bcl-xL. In addition, furanodiene treatment caused the up-regulation of tumor necrosis factor receptor 1 (TNFR1), the formation of TNFR1 complex and an obvious production of TNF-α in HL60 cells. The soluble TNFR1 receptor effectively inhibited furanodiene-induced apoptosis. Collectively, these results evidenced that furanodiene could inhibit leukemia cells growth via apoptotic pathway, and TNFR1-mediated extrinsic apoptotic pathways explains furanodiene-induced apoptosis [281]. Furanodiene inhibited HepG2 cell growth by causing cell cycle arrest at G2/M and inducing apoptosis as evidenced by DNA fragmentation assay. This terpene induced mitochondrial transmembrane depolarization, release of mitochondrial cytochrome c, activation of caspases-3 and the cleavage of PARP. The activation of p38 and the inhibition of ERK mitogen-activated protein kinase (MAPK) signaling were also demonstrated [282].

### 4.4. European and American Pharmacopoeias

Over 3000 plants are sources of essential oils, though only around 300 of these are of commercial value. Approximately 30 of these latter plants, often cultivated on a large scale, produce essential oils whose use as drugs dates back centuries in different cultures throughout the world, and are described in international and national Pharmacopoeias. In Table 2, the essential oils included in European and American Pharmacopoeias are listed.

#### 4.4.1. Preclinical Studies

Literature data published over the last five years (from 2011 up to June 2016) regarding the pharmacological properties of essential oils from *Eucalyptus*, *Mentha*, and *Citrus* species, are reported in the following paragraphs. These essential oils were chosen based on the number of studies reported in the PubMed database. In fact, the literature search performed in PubMed using the keywords “*Eucalyptus* species essential oil”, “*Mentha* species essential oil”, and “*Citrus* species essential oil” each produced about of 500 articles, while other essential oils have been studied less.

##### *Eucalyptus* Species Essential Oils

In recent years, the essential oils extracted from the *Eucalyptus* species have been of great interest for their potential use as drugs against infectious diseases, due to a high content of health-promoting bioactive compounds with antimicrobial activity.

In 2012, Bachir et al. [282] demonstrated that the essential oil of the leaves of *E. globulus* Labill. obtained through the hydrodistillation method (using a Clevenger-type apparatus) showed antibacterial activity against clinically isolated strains of *Escherichia coli* and *Staphylococcus aureus* by using agar disc diffusion and broth dilution methods. The diameter of the inhibition zone ranged from 8 to 26 mm. The largest diameter was registered on *E. coli* treated with pure essential oil, while *S. aureus* was found to be more sensitive than *E. coli* at the lowest concentrations. The authors concluded that *E. globulus* leaf essential oil could be used as a natural antibiotic for the treatment of several Gram-positive and Gram-negative infectious diseases.

In the same year, Döll-Boscardin et al. [283] published a paper on the in vitro cytotoxic activity of *E. benthamii* Maiden & Cambage leaf essential oil (extracted from air dried young and old leaves by hydrodistillation in a Clevenger-type apparatus). In addition, some terpenes occurring in these essential oils were studied (α-pinene, terpinen-4-ol, and γ-terpinene). Cytotoxicity was tested on human and experimental animal cancer cell lines (Jurkat, which are T leukemia cells, J774A.1, from murine macrophage tumor, and HeLa from cervical cancer) using a 3-(4,5-dimethylthiazol-2-yl)-2,5-diphenyltetrazolium bromide (MTT) assay. Moreover, the chemical compositions of the two samples were determined by gas chromatography/mass spectrometry (GC-MS), showing that the concentrations of monoterpene hydrocarbons and oxygenated sesquiterpenes were similar for essential oils obtained from either young or old leaves, while the sesquiterpene hydrocarbon and oxygenated monoterpene concentrations were higher in the essential oil extracted from young leaves than that obtained from old leaves. The results of the in vitro MTT test showed that these essential oils were more active than the isolated terpenes. Jurkat and HeLa cell lines were more sensitive to the essential oils than the murine cancer cell line, suggesting that *E. benthamii* leaf essential oils can be used as cytotoxic agents and potential anticancer drugs.

Elaissi et al. [284] extended their study to antimicrobial activity, also investigating the antifungal and antiviral properties of eight essential oils (obtained by hydrodistillation following the European Pharmacopoeia recommendations) from eight *Eucalyptus* species grown in Tunisia (*E. bicostata* Maiden, Blakely & Simmonds, *E. cinerea* F. Muell. ex Benth., *E. maidenii* F. Muell., *E. odorata* behr, *E. sideroxylon* A. Cunn. ex Wollss, *E. astringens* (Maiden) Maiden, *E. lehmannii* (Schauer) Benth. and *E. leucoxylon* F. Muell.). In this case, the antibacterial activity was studied against a large number of clinical bacterial isolates (*Haemophilus influenza*, 11 strains; *Klebsiella pneumoniae*, 13 strains; *Pseudomonas aeruginosa*, 10 strains; *S. aureus*, 17 strains; *Streptococcus agalactiae*, nine strains; *Streptococcus pneumoniae,* 19 strains; and *Streptococcus pyogenes*, two strains) using paper-disc agar and diffusion methods. The results showed that the mean diameter of inhibition for all essential oils ranged from 27.4 to 6.0 mm, while the MIC values ranged from 0.30 to 169.00 mg/mL for the most active samples. The antifungal activity was tested against five fungal strains including *Candida albicans*, *Scopulariopsis brevicaulis*, *Trichophyton rubrum*, *Trichophyton soudanense*, and *Microsporum canis*, using the agar incorporation method. The percentage of inhibition at a concentration of 1000 ppm was between 0.1%–100%, depending on the *Eucalyptus* species and the fungal strain. Finally, the antiviral activity was studied using Confluent Vero cell cultures that were treated with non-cytotoxic concentrations of the essential oils both during and after viral infection. The tested essential oils showed different activities here too, with oils from *E. sideroxylon*, *E. lehmannii*, *E. leucoxylon* and *E. odorata* having no antiviral activity, while *E. bicostata* (IC_50_ = 0.7–4.8 mg/mL), *E. astringens*, and *E. maidennii* (IC_50_ = 136.5–233.5 mg/mL) being the most active against viral infections. The chemical analyses performed, using GC-MS and GC/flame ionization (GC-FID) methods, led to the identification of 25 components (among which 1,8-cineole, cryptone, α-pinene, *p*-cymene, and α-terpineol occurred in concentrations above 10%).

A more recent in vitro investigation, carried out on the air-dried leaves of *E. gunnii* Hook. f. submitted to hydrodistillation according to *Eur. Pharm*. *4* [285], focused on antioxidant, antimutagenic and antibacterial properties. The antioxidant activity was determined on the stable 2,2-diphenyl-1-picrylhydrazyl (DPPH) radical. The results showed that the radical scavenging capacity of *E. gunnii* essential oil was significantly lower than that of synthetic antioxidant compounds. The antimutagenic activity was tested against spontaneous and *t*-BOOH-induced mutagenesis in *E. coli* IC202 *oxyR*, a strain deficient in removing ROS, which was treated with subtoxic essential oil concentrations ranging from 0.05 to 0.15 µL/plate. The inhibition of spontaneous mutagenesis in the presence of the essential oil was modest (12% at the highest tested concentration). The essential oil showed higher antimutagenic activity in the presence of *t*-BOOH-induced mutagenesis, 18% at the lowest concentration and 23% at the highest. Finally, the antibacterial activity was determined against *S. aureus* ATCC25923, *Staphylococcus epidermidis* ATCC12228, *Pseudomonas aeruginosa* ATCC27853, *E. coli* ATCC25922, *Bacillus subtilis* ATCC10774, *Micrococcus flavus* ATCC10240, *Klebsiella pneumoniae* NCIB9111 and *E. coli* SY252 and IB112 strains, using the dilution method and the disc-diffusion assay (chloramphenicol 30 μg/disc, streptomycin 100 μg/disc, bacitracin 0.04 IU/disc, and gentamycin 40 μg/disc were used as positive controls). The antibacterial activity, expressed as the diameter of the growth inhibition zone and MIC resulted to be modest. In fact, *E. gunnii* essential oil only showed antibacterial activity on *S. epidermidis* and *B. subtilis* under the disc-diffusion assay (at concentrations higher than 1.66 μL/mL) and on *M. flavus*, *K. pneumoniae*, and *E. coli lpc*A under the dilution method (MIC 0.83 mg/mL). The essential oil was submitted to GC-MS analysis for the determination of its chemical composition, and the most concentrated compounds were found to be 1,8-cineole (67.80% dry matter) followed by α-pinene (14.12%), β-phellandrene (3.92%), α-terpinyl acetate (3.27%), *trans*-pinocarveol (2.49), and α-terpineol (2.08%), with the other constituents found at concentrations below 1% [286].

In the last two years, three more research articles have been published on the chemical composition and antibacterial activity of the essential oils extracted from *Eucalyptus* species, with similar findings. In more detail, Sebei et al. [287] have expanded on the investigation of Tunisian essential oils obtained from the leaves of Eucalyptus species, studying its chemical composition and antibacterial activity. In comparison with previous investigations, they demonstrated its antibacterial activity against *Enterococcus faecalis*, *Listeria ivanovii*, and *Bacillus cereus*. Mekonnen et al. [288] studied the antibacterial and antifungal properties of *E. globulus* essential oil against *Salmonella* and *Shigella* species, and *Trichophyton* and *Aspergillus* species. The antifungal activity of the *E. smithii* F. Muell. ex R. T. Baker essential oil was also investigated by Baptista et al. [289] showing that this essential oil is active against several dermatophytes (*Microsporum canis* ATCC 32903, *M. gypseum* ATCC 14683, *Trichophyton mentagrophytes* ATCC 9533, *T. mentagrophytes* ATCC 11480, *T. mentagrophytes* ATCC 11481, and *T. rubrum* ATCC 5507), suggesting a potential use for the treatment of dermatophytosis.

In 2016, Knezevic et al. [70] showed that essential oil from *E. camaldulensis* Dehnh. is active against *Acinetobacter baumannii*, which is an emerging multidrug-resistant pathogen diffused worldwide. The anti-melanogenic activity of the essential oil of this plant has also been studied by means of spectrophotometric determination of the melanin content of mouse melanoma cells and cellular tyrosinase activities, and Western blotting for the evaluation of the levels of expression of melanogenesis-related proteins, after treatment with non-toxic concentrations of the essential oil extracted by hydrodistillation from the flowers of *E. camaldulensis*. In the same study, the authors investigated antioxidant activity, using DPPH scavenging activity and 2,2′-azino-bis(3-ethylbenzothiazolin-6-sulphonic acid) (ABTS) scavenging capacity assays to explain the potential mechanisms of action at the basis of the anti-melanogenic activity. In addition, cellular ROS levels were registered. The results showed that essential oil extracted from *E. camaldulensis* flowers was able to inhibit melanogenesis through its antioxidant capacity and the down-regulation of mitogen-activated protein kinase (MAPK) and protein kinase A (PKA) signaling pathways. Therefore, the authors concluded that this plant product could be used in the treatment of skin diseases [290].

As regards in vivo studies, in 2013, Gbenou et al. [291] investigated the anti-inflammatory and analgesic properties of *E. citriodora* Hook. essential oil on Wistar rats fed with food mixed with this essential oil at doses ranging from 600 to 2600 mg/kg. The most abundant components of this oil were citronellal (83.50%) followed by isopulegol (4.40%) and methyleugenol (2.20%). The analgesic properties were determined using tail flick and tail immersion Koster methods. Anti-inflammatory activity was assessed by injecting 0.1 mL of 1% (*v*/*v*) formol into the aponeurosis of the left foot of the rats to cause an edema. The results showed that *E. citriodora* essential oil exerted in vivo analgesic and anti-inflammatory activities, suggesting a potential use as adjuvant therapeutic agent against inflammatory-related diseases.

##### *Mentha* Species Essential Oils

Over the last five years, the essential oils extracted from the leaves and flowers of *Mentha* species have been studied for their antimicrobial and anti-inflammatory properties.

In 2012, Saharkhiz et al. [292] evaluated the antifungal and antibiofilm activities of the essential oil prepared by hydrodistillation of the aerial parts of *Mentha* x *piperita* L., collected during the full flowering stage. The essential oil was submitted to GC-MS analysis to determine its chemical composition, revealing that the most concentrated components were menthol (53.28%), menthyl acetate (15.10%), menthofuran (11.18%), 1,8 cineole (6.69), neomenthol (2.79%), menthone (2.45%), (*Z*)-caryophyllene (2.06%), germacrene D (2.01%) and numerous other substances which occurred in concentrations below 2%. The antifungal activity of this essential oil was investigated against 25 standard strains of fungi (including *Candida* and *Aspergillus* species), and 35 clinical isolates of yeasts, and the capacity to inhibit growth and biofilm formation, which is an important aspect for fungal survival and a mechanism of drug resistance. Antifungal activity, determined by means of the microdilution method using fluconazole as a positive control, was expressed as MIC and minimum fungicidal concentration (MFC). The essential oil showed high fungistatic activity against all tested strains with MIC values ranging from 0.5 to 8 µL/mL for *Candida* and 0.5 to 4 µL/mL for *Aspergillus* species, respectively. Moreover, it showed fungicidal activity against encapsulated yeasts such as *C. neoformans*, responsible for meningitis. In addition, the essential oil inhibited the formation of biofilms by *C. albicans* at a concentration of 2 µL/mL.

These results have been confirmed by a more recent study published by Ibrahim et al. which showed that several essential oils, and among these the essential oil of fresh mint leaves (*M. piperita*), possess antimycotic activity against clinical keratinophilic fungi (*Microsporum canis*, *Epidermophyton floccosum*, *Trichophyton rubrum*, and *Trichophyton mentagrophytes*) isolated from patients with superficial fungal infections, showing a MFC ranging from 2 to 4 µL/mL [288]. In the same year, another study showed that a commercial sample of *M. piperita* essential oil, as well as several standard compounds occurring in the oil (carvone, menthol, and menthone), exerted anti-candida activity, with the essential oil and carvone being most active with MIC values of 225 mg/mL and 248 mg/mL, respectively. The mechanism of action was investigated by assessing (1) the enzymatic activity of plasma membrane H^+^ ATP-ase (which maintains ion balance and is crucial for *Candida* nutrient uptake and morphogenesis); (2) H^+^ efflux mediated by plasma membrane H^+^ ATP-ase; (3) ergosterol biosynthesis; and (4) the ultrastructure of *Candida* cells, by means of scanning electron microscopy. At MIC values, the essential oil and its constituents induced a reduction in enzymatic activity of about 50%. Moreover, a decrease in ergosterol content, cell membrane damage, and modifications in *Candida* cell morphology were registered, suggesting that the antifungal activity of *M. piperita* essential oil has a multi-factorial mechanism [117]. In the same year, Hussain et al. confirmed the anti-biofilm activity of *M. piperita* essential oil against Gram-negative bacteria (such as *Chromobacterium violaceum*, *Pseudomonas aeruginosa*, and *Aeromonas hydrophila*). The antibacterial activity the oil and its effects on virulence factors of *P. aeruginosa* and *A. hyrophila* (LasB elastase, protease, pyocyanin, chitinase, swarming motility, EPS extraction) were studied, showing the capacity of this essential oil and menthol to interfere with the quorum sensing systems of tested Gram-negative bacteria. These findings are interesting because they show that an essential oil can influence the systems used by bacteria to coordinate gene expression according to the density of the microbial population, including the production of virulence factors [63].

Mint essential oils are also well-known for their antioxidant activity. Sun et al. [293] conducted an interesting research on the essential oil extracted from the leaves of Chinese *M. piperita*, assessing its in vitro and in vivo anti-inflammatory activity, in vitro cytotoxic activity against cancer cell lines, and in vitro antioxidant activity. The anti-inflammatory activity was tested in a male mouse model, in which croton oil induced right ear edema in the experimental animals, topically treated with the essential oil (200, 400, and 800 mg/ear) or indomethacin (used as a positive control at the doses of 300 mg/ear) about 60 min before the croton oil treatment. The evaluation of the anti-inflammatory activity was tested by comparing the weight of the left (control) and right (treated) ear. The results showed that the essential oil decreased ear edema by 5.77%, 7.37%, and 30.24% at doses of 200, 400, and 800 mg, respectively, showing a similar activity to that given by the indomethacin treatment, which reduced the edema by 16.79%. These results were also confirmed in in vitro conditions. In RAW 264.7 cells stimulated with LPS and treated with the essential oil, a decrease in the production of NO and PGE2, well-known mediators in acute inflammatory responses, was registered. In the same investigation, the cytotoxic effect of *M. piperita* essential oil against cancer cell lines (human lung carcinoma SPC-A1 cells, human gastric cancer SGC-7901 cells, human leukemia K562 cells and human hepatocellular carcinoma BEL-7402 cells) was determined by MTT assay 24 h after treatment. The cytotoxic activity was found to be dependent on the cell line. In particular, K562 and SGC-7901 cells were found to be the most sensitive to the cytotoxic effects of this essential oil, with IC_50_ being 16.16 mg/mL and 38.76 mg/mL, respectively. Finally, the authors studied the in vitro antioxidant activity with different common methods such as DPPH antiradical activity, reducing power, and anti-hydroxyl radical activity, showing that the tested essential oil exerted a moderate antioxidant capacity. The chemical composition of the essential oil was again determined via GC-MS, allowing the identification of 51 compounds, with menthol (30.69%), menthone (14.51%), menthyl acetate (12.86%), and neomenthol (9.26%) accounting for more than 50% of the constituents occurring in the essential oil.

##### *Citrus* Species Essential Oils

*Citrus* is the largest genus of the Rutaceae family, including about 70 species. *Citrus aurantium* L. (common name: bitter orange), *Citrus bergamia* Risso & Poit. (which is a synonym of *Citrus limon* (L.) Osbeck (common name: bergamot)), *Citrus paradisi* Macfad. (common name: grapefruit) and *Citrus limon* (L.) Osbeck (common name: lemon) are the most common sources of essential oils. Therefore, this section will focus on the essential oils obtained from these species, which are widely under study for their broad spectrum physiological and pharmacological properties including antimicrobial, anti-inflammatory, analgesic, antiproliferative, antimicrobial, and antioxidant activities.

As regards antimicrobial activity, Furneri et al. [294] published a research article, in 2012, on the antimycoplasmal activity of *C. bergamia* essential oil and its main constituents (limonene, linalyl acetate and linalool). The antibacterial activity was tested against 42 strains of *Mycoplasma hominis*, two strains of *Mycoplasma fermentans* and a strain of *Mycoplasma pneumoniae*. The MIC values registered for the essential oil ranged from 0.5 to 1% (*v*/*v*), with linalool and limonene showing high activity, especially on *M. pneumoniae* (MIC values 0.015% and 0.03%, respectively). In the same year, Adukwu et al. [295] studied the antibacterial activity and anti-biofilm property of several essential oils, including some obtained from *Citrus* species (grapefruit and bergamot). The antimicrobial activity of the *Citrus* species essential oils was confirmed against *S. aureus.* Moreover, the authors found that grapefruit essential oil did not show any anti-biofilm activity, unlike other essential oils, such as that extracted from lemongrass (*Cymbopogon flexuosus* (Nees ex Steud.) W. Watson).

*C. aurantium* leaf essential oil showed antimicrobial activity, too. In fact, the essential oils prepared from leaves collected in different seasons were found to exert high activity against some Gram-positive bacteria (*B. subtilis* with a MIC value of 2.7 mg/mL, and *S. aureus* with a MIC value of 4.8 mg/mL), and moderate activity against yeasts and fungi (*Saccharomyces cerevisiae*, with a MIC value of 9.2 mg/mL and *Mucor ramannianus*, with a MIC value of 5 mg/mL). Chemical analysis performed through GC-MS allowed the identification of 46 compounds, with the highest concentrations of linalool (43.2% to 65.97%), linalyl acetate (0.77% to 24.77%), and α-terpineol (9.29% to 12.12%) [296]. Similar results have been obtained by Hsouna et al. [297] and Ammar et al. [298] more recently, reporting the antimicrobial activity of the essential oil obtained from the flowers of *C. aurantium.* In addition, the antifungal activity of *C. aurantium* essential oil was investigated [299].

In recent years, *Citrus* x *limon* essential oil has been widely studied for its chemical composition and antimicrobial activity. Hamdan et al. [300] investigated the volatile metabolites extracted from the essential oils of fruit peel and leaves by GC-MS. Among the 141 identified components, limonene is more concentrated in fruit peel essential oil (52.73%) than in leaf oil (29.13%). Moreover, γ-terpinene (9.88%), β-pinene (7.67%), geranial (4.44%), and neral (3.64%) were found to be the main constituents of fruit peel oil, while neral (12.72%), neryl acetate (8.53%), *p*-menth-1-en-7-al (4.63%), β-pinene (6.35%), and nerol (4.42%) are the main substances occurring in leaf essential oil. The same research showed that lemon oil exerts good antimicrobial activity against Gram-positive and Gram-negative bacteria, and yeasts (i.e., *B. subtilis*, *Staphylococcus capitis*, *Micrococcus luteus*, *Pseudomonas fluorescens*, *S. cerevisiae,* and *Candida parapsilosis*). In 2014, Settanni et al. [301] studied the influence of seasons on the chemical composition of three different cultivars of lemon used to prepare essential oil. The main results focused on the chemical composition and the antibacterial activity that were found to vary according to both the season and the cultivar.

With regards to the anti-inflammatory activity of *Citrus limon* oil, Mitoshi et al. [302] assessed the in vitro antiallergic and anti-inflammatory properties of 20 essential oils, one of which was a cold-pressed lemon essential oil. In more detail, the authors determined the release of β-hexosaminidase from rat basophilic leukemia (RBL-2H3) cells treated with the calcium ionophore, A23187. For the anti-inflammatory activity, they evaluated the reduction of tumor necrosis factor-α (TNF-α) in RAW264.7 murine macrophages, in which inflammation was induced by a lipopolysaccharide (LPS) treatment. Among tested essential oils, lemon oil showed high activity, though lower than that registered for lemongrass oil.

The anti-inflammatory activity of essential oils may be the basis of their protective activity against gut inflammation diseases. Polo et al. [303] studied the effects of essential oil prepared from the fruit peel of *C. aurantium* on a model which accurately reflects human gastric ulcer, involving male Wistar rats in which a gastric lesion was induced via acetic acid. The results showed that bitter orange oil significantly decreased the gastric lesion area by 76% compared to the control group, at an oral dose of 250 mg/kg/day for 14 consecutive days.The protective effect of this essential oil was confirmed by the same research group, which published a study on the effect of d-limonene (one of the main compounds occurring in bitter orange essential oil) in the same gastric ulcer model system. The same treatment reported in their previous investigation induced an increase in cell proliferation and cyclooxygenase 2 expression in the gastric mucosa, as well as vascular endothelial growth factor (VEGF)-mediated blood vessel formation. In addition, the treatment caused the over-production of gastric mucus, which plays a role in increasing the gastric barrier. The authors concluded that, considering the low adverse and side effects of this essential oil, it could be considered a good candidate for gastric ulcer treatment [303].

In 2014, Bonamin et al. [304] demonstrated the protective activity of β-myrcene, another monoterpene occurring in *C. aurantium* oil, orally administered at a dose of 7.50 mg/kg for 14 consecutive days to experimental animals in a different model of gastric ulcer (induced by ethanol, non-steroidal anti-inflammatory drugs, *Helicobacter pylori*, stress, ischemia-reperfusion injury, cysteamine). Besides the capacity to decrease gastric and duodenal lesions and the production of mucus, β-myrcene was found to be able to increase mucosal lipid peroxidation (registered through an increase in malondialdehyde levels) and antioxidant defenses (glutathione peroxidase, glutathione reductase, and total glutathione in gastric tissues), thus suggesting that oxidative stress plays an important role in the activity of β-myrcene.

The influence of bergamot essential oil on in vitro oxidative stress was also studied by Cosentino et al. [305], which used human polymorphonuclear leukocytes, isolated from venous blood of healthy volunteers, as a cell model system. Human polymorphonuclear leukocytes play an essential role in innate immunity as defense against microbial infections, and in inflammation as sources of various pro-inflammatory cytokines. To measure the intracellular ROS levels, the cells were treated with the redox-sensitive dye 2,7-dichlorofluorescein diacetate. The influence of bergamot oil was evaluated on cells treated with *N*-formyl-Met-Leu-Phe (at a concentration of 0.1 μM), which is a chemotactic peptide that reacts with membrane receptors stimulating ROS production, and phorbol myristate acetate (at a concentration of 0.1 ng/mL), which stimulates the intracellular protein kinase C. In addition, cells were grown in the presence of extracellular or intracellular Ca^2+^ chelators. The results showed that bergamot essential oil (0.1%) increased intracellular ROS production induced by *N*-formyl-Met-Leu-Phe, in presence of extracellular Ca^2+^, while reducing the effect of protein kinase C activator phorbol myristate acetate (at concentrations ranging from 0.03 to 0.1%). The authors concluded that the pro-inflammatory potential of the essential oil needs to be carefully considered for any future clinical applications.

Different results have been achieved by Loizzo et al. [306] which studied the essential oils extracted from lemon (*Citrus x limon*) fruit peels and leaves using different extraction methods (hydrodistillation, supercritical fluid extraction SFE, and Soxhlet extraction). The essential oils exerted different antioxidant and radical scavenging activities in in vitro chemical assays (DPPH, ABTS, ferric reducing ability power FRAP, and β-carotene bleaching test), depending on the part of the fruit (peels or leaves) and the extraction technique. The essential oils were also submitted to chemical analysis by GC-MS, showing that the most abundant component was limonene (ranging from 14.08% to 59.64%), followed by γ-terpinene (ranging from 1.82% to 19.03%), and β-pinene (ranging from 3.24% to 17.10%). The authors showed that the monoterpene hydrocarbon fraction was positively correlated in all antioxidant assays, suggesting that this fraction could be considered partly responsible for the observed antioxidant activity.

Finally, essential oils from *Citrus* species have been investigated for their potential anticancer activity. In fact, in 2013, two studies were published on the cytotoxicity of bergamot oil on a human neuroblastoma cell line. In more detail, the first study by Celia et al. [307] investigated the cytotoxic activity of bergamot oil, both alone and loaded into pegylated liposomes, on neuroblastoma cells (SH-SY5Y) through MTT test. The idea of studying the effect of essential oil liposomes arose from the consideration that many studies showed the cytotoxic activity of these essential oils, however their high lipophilicity, poor water solubility, low stability and thus low bioavailability put limitations on their use as therapeutic agents. New forms of delivery, such as liposomes, could increase the bioavailability of the essential oil and, therefore, its potential use in cancer therapy. The results showed that bergamot oil liposomes possessed higher activity than raw essential oil, being able to decrease SH-SY5Y cell viability at concentrations (0.01%–0.02% *v*/*v*) lower than those registered for the essential oil (0.02%–0.04% *v*/*v*). The authors emphasized the potential role of new delivery systems in improving the cytotoxic activity of essential oils.

Russo et al. [308] focused their investigation on compounds responsible for the anti-proliferative activity of bergamot oil. Limonene, linalyl acetate, linalool, γ-terpinene, β-pinene and bergapten were individually tested in neuroblastoma cell cultures (SH-SY5Y) at the same concentrations in which they occur in the essential oil. Interestingly, the tested compounds did not induce cell death, whilst significant cytotoxicity was registered in cell cultures treated with a mixture of limonene and linalyl acetate. These results were confirmed only for this mixture, which was able to influence both morphological and biochemical parameters such as caspase-3 activation, PARP cleavage, DNA fragmentation, cell shrinkage, and cytoskeletal alterations. In conclusion, though it may be very difficult to compare the results summarized above, due to the fact that the data have been obtained through a variety of methods, with different amounts of essential oils/compounds tested against a range of microbial strains, cancer cell lines and experimental animal models, the findings of the reported studies may be considered as a basis for detailed investigation of the in human activity of essential oils.

#### 4.4.2. Clinical Studies

A second literature search was conducted on the ClinicalTrials.gov database [309], using the keywords: “*Eucalyptus* essential oils”, “*Mentha* essential oil”, and “*Citrus* essential oil”. This database reported six completed studies on *Eucalyptus* essential oils (Table 3). These studies were carried out between 2008 and 2016 and focused on two main issues: the effects of *Eucalyptus* essential oil inhalation on cardiovascular system and respiratory tract. None of these clinical trials posted their results or indicated publications regarding these studies.

As regards *Mentha* species, eight studies have been reported, among which four are completed clinical trials conducted between 2007 and 2016 (Table 4). Four studies focused on the potential effects of mint essential oil on oral health including gingivitis, dental plaque accumulation, and the reduction in bacterial counts through the use of a mouthwash. The results showed that Cool Mint Listerine^®^ antiseptic mouth rinse (20 mL), twice daily after brushing, induced a modified whole mouth gingival index (MGI) of 2, in comparison with a 5% hydroalcoholic mouth rinse (20 mL, negative control) and Listerine Zero^®^ mouth rinse (20 mL) twice daily after brushing. Three studies evaluated the influence of the consumption of a dietary supplement on respiratory tract diseases (rhino-sinusitis, viral pharyngitis, viral tonsillitis, and viral tracheitis). Finally, one clinical trial studied the effect of ice pops to alleviate pregnancy related nausea and vomiting (morning sickness).

Finally, with regard to *Citrus* essential oil, six clinical trials have been registered, with two completed studies carried out in 2009 and 2011 (Table 5). Two studies focused on distress, one on morning sickness and three on the potential beneficial effects on cancer (uterine cervical dysplasia, papillomavirus infections, breast cancer and the symptoms of chemotherapy). The completed clinical trials on distress (anxiety and depression) showed that lemon oil increased positive mood compared to water and lavender oil, which is known for its relaxant effect. In addition, the levels of norepinephrine following the cold pressor were constant when subjects smelled lemon, compared to water or lavender. The inhalation of essential oils did not modify parameters related to inflammation and the immune-system (IL-6 and IL-10, salivary cortisol, heart rate, blood pressure) following the cold pressor. The limited data on the in human efficacy of essential oils, as well as controversies on their mechanisms of action lead to the conclusion that further studies are required to reach a definitive recommendation on the use and beneficial effects of essential oils in human healthcare.

## 5. Conclusions

Among natural plant products, essential oils deserve particular attention because of their uses in many different traditional healing systems all over the world. In addition, currently, distillation of essential oils from plant organs is a highly reliable and affordable process.

As regards their efficacy, a huge amount of preclinical studies have documented the biological activities of essential oils, also elucidating their mechanism of action and pharmacological targets. In particular, antimicrobial, antioxidant, anti-inflammatory and anticancer activities have been demonstrated in a number of cell and animal models. However, the paucity of in human studies, compared with the in vivo/in vitro ones, limits the potential of essential oils as effective and safe phytotherapeutic agents, though the efficacy of essential oils in oral healthcare is well documented [310]. Therefore, more well-designed clinical trials are needed in order to reach a high level of scientific evidence and ascertain the real efficacy and safety of plant products which have accompanied humans since ancient times.

## Figures and Tables

**Table 1:** Preclinical anticancer activity of essential oils from medicinal plants commonly used in Traditional Korean Medicine.

Family	Scientific Name	Tumor (Cancer Cell Lines)	References
Amaryllidaceae	*Allium sativum* L.	Human breast, colon, lung, skin and liver cancers	[246]
Burseraceae	*Boswellia serrata* Roxb. ex Colebr.	Human colon carcinoma (HCT116)	[247]
Compositae	*Artemisia capillaris* Thunb.	Human oral epidermal carcinoma (KB)	[95]
	*Artemisia iwayomogi* Kitam.	Human oral epidermal carcinoma (KB)	[248]
	*Artemisia lavandulaefolia* Nakai	Human cervix adenocarcinoma (HeLa)	[249]
Pinaceae	*Pinus koraiensis* Siebold & Zucc.	Human colon carcinoma (HCT116)	[93]
Solanaceae	*Solanum nigrum* L.	Liver, mammary, uterine cervix and gastric cancers	[250]
	*Solanum spirale* Roxb.	Human breast (MCF-7) and small cell lung (NCI-H187) cancers, human oral epidermal carcinoma (KB)	[251,252]
	*Solanum stipulaceum* Roem. & Schult	Human acute promyelocytic leukemia (HL-60) and acute monocytic leukemia (THP-1)	[253]
	*Solanum erianthum* D. Don	Human breast carcinoma (Hs 578T) and prostate carcinoma (PC-3)	[254]
	*Solanum macranthum* Dunal	Human breast carcinoma (Hs 578T) and prostate carcinoma (PC-3)	[254]
Verbenaceae	*Vitex rotundifolia* L.f.	Breast cancer (MCF-7)	[255]
Zingiberaceae	*Curcuma longa* L.	Human oral epidermal carcinoma (KB), murine leukemia (P388), leukemia (HL-60, K-562 and L1210)	[256,257,258,259]
	*Curcuma zedoaria* Roxb.	Mouse melanoma (B16BL6), human hepatoma (SMMC-7721), non-small cell lung carcinoma (NSCLC)	[91,260]
	*Curcuma wenyujin* Y.H.Chen & C.Ling	Cervical, liver (HepG2), lung and gastric (AGS and MGC 803) cancers	[261,262,263]

**Table 2:** Essential oils extracted from a variety of plants listed in European and American Pharmacopoeias.

Essential Oil Name	Botanical Name	Plant Part Used	European Pharmacopoeias	American Pharmacopoeias
Anise oil	*Pimpinella anisum* L.	ripe fruit	X	X
Bitter-fennel fruit oil	*Foeniculum vulgare* Mill., spp. *vulgare* var. *vulgare*	ripe fruit	X	X
Bitter-fennel herb oil	*Foeniculum vulgare* Mill., spp. *vulgare* var. *vulgare*	aerial parts	X	
Caraway oil	*Carum carvi* L.	dry fruit	X	X
Cardamom oil	*Elettaria cardamomum* L.	seeds		X
Cassia oil	*Cinnamomum aromaticum* Nees	leave and young branches	X	
Cinnamon bark oil, Ceylon	*Cinnamomum zeylanicum* Nees	bark of the shoot	X	
Cinnamon leaf oil, Ceylon	*Cinnamomum zeylanicum* Nees	leaves	X	
Citronella oil	*Cymbopogon winterianus* Jowitt ex Bor	fresh or partially dried aerial parts	X	
Clary sage oil	*Salvia sclarea* L.	fresh or dried flowering stem	X	
Clove oil	*Syzygium aromaticum* (L.) Merr. & L.M.Perry	dried flower buds	X	X
Coriander oil	*Coriandrum sativum* L.	fruits	X	X
Dwarf pine oil	*Pinus mugo* Turra	fresh leaves and twigs	X	
Eucalyptus oil	genus *Eucalyptus*	fresh leaves or fresh terminal branches of various species of *Eucalyptus* rich in 1,8-cineole	X	
Juniper oil	*Juniperus communis* L.	ripe, non-fermented berry cones	X	
Lavender oil	*Lavandula angustifolia* Mill.	flowering tops	X	
Lemon oil	*Citrus limon* L.	fresh peel	X	X
Mandarin oil	*Citrus reticulata* Blanco	fresh peel	X	
Matricaria oil	*Matricaria recutita* L.	fresh or dried flower-heads or flowering tops	X	
Mint oil (partly dementholised by cristallization)	*Mentha canadensis* L.	fresh, flowering aerial parts	X	
Neroli oil	*Citrus aurantium* L.	fresh flowers	X	
Nutmeg oil	*Myristica fragrans* Houtt.	dried and crushed kernels	X	
Peppermint oil	*Mentha piperita* L.	fresh aerial parts of the flowering plant	X	X
Pine sylvestris oil	*Pinus sylvestris* L.	fresh leaves and branches	X	
Rose oil	*Rosa gallica* L., *Rosa damascene* Miller, *Rosa alba* L., *Rosa centifolia* L.	fresh flowers		X
Rosemary oil	*Rosmarinus officinalis* L.	flowering aerial parts	X	
Spanish sage oil	*Salvia lavandulifolia* Vahl	flowering aerial parts	X	
Spike lavender oil	*Lavandula latifolia* Medik	flowering tops	X	
Star anise oil	*Illicium verum* Hook	dry ripe fruits	X	
Sweet orange oil	*Citrus sinensis* (L.) Osbeck	fresh peel	X	X
Tea tree oil	genus *Melaleuca*	foliage and terminal branches	X	
Thyme oil	*Thymus vulgaris* L. or *Thymus zygis* L.	fresh flowering aerial parts	X	
Turpentine oil, Pinus pinaster type	*Pinus pinaster* Aiton	oleoresin obtained by tapping	X	

**Table 3:** Clinical trials registered on the Clinicaltrials.gov database conducted on *Eucalyptus* essential oil.

“*Eucalyptus* Essential Oil” Clinical Trials	
**Study Title**	**Study to Investigate Effects of Different Therapies for the Treatment of Hypertension**
Condition	Hypertension
Status	Completed
Start and Completion dates	March 2014–July 2015
Study Type and Design	Interventional
	Allocation: Randomized
	Endpoint Classification: Efficacy Study
	Intervention Model: Crossover Assignment
	Masking: Open Label
	Primary Purpose: Treatment
Primary Outcome	Blood pressure
Condition	Hypertension
Status	Completed
Start and Completion dates	August 2013–April 2014
Study Type and Design	Interventional
	Allocation: Randomized
	Intervention Model: Crossover Assignment
	Masking: Single Blind (Subject)
Primary Outcome	Blood pressure response to Inhalation of Essential Eucalyptus Oil
Study Title	**Treatment of Acute Rhino-Sinusitis With Essential Oils of Aromatic Plants**
Condition	Rhino-Sinusitis
Status	Completed
Start and Completion dates	January 2008–May 2008
Study Type and Design	Interventional
	Allocation: Randomized
	Endpoint Classification: Efficacy Study
	Intervention Model: Parallel Assignment
	Masking: Double Blind (Subject, Investigator)
	Primary Purpose: Treatment
Primary Outcome	To demonstrate a relief in the nasal obstruction within the first 20 min after first administration of treatment with the spray
Study Title	**Treatment of Acute Pharyngo-Tonsillitis with Essential Oils of Aromatic Plants**
Condition	Viral Pharyngitis—Viral Tonsillitis
Status	Completed
Start and Completion dates	January 2008–May 2008
Study Type and Design	Interventional
	Allocation: Randomized
	Endpoint Classification: Efficacy Study
	Intervention Model: Parallel Assignment
	Masking: Double Blind (Subject, Investigator)
	Primary Purpose: Treatment
Primary Outcome	To demonstrate a throat pain relief within the first 20 min after first administration of treatment with the spray
Study Title	**Treatment of Acute Tracheitis and Laryngitis with Essential Oils of Aromatic Plants**
Condition	Viral Laryngitis—Viral Tracheitis
Status	Completed
Start and Completion dates	January 2008–May 2008
Study Type and Design	Interventional
	Allocation: Randomized
	Endpoint Classification: Efficacy Study
	Intervention Model: Parallel Assignment
	Masking: Double Blind (Subject, Investigator)
	Primary Purpose: Treatment
Primary Outcome	To demonstrate a cough or hoarseness relief within the first 20 min after first administration of treatment with the spray
Study Title	**Study on Hypertonic Saline Nasal Spray (PhytosunDecon)**
Condition	Nasal Congestion
Status	Completed
Start and Completion dates	March 2014–October 2014
Study Type and Design	Interventional
	Allocation: Randomized
	Endpoint Classification: Pharmacokinetics Study
	Intervention Model: Parallel Assignment
	Masking: Double Blind (Subject, Caregiver)
	Primary Purpose: Treatment
Primary Outcome	Assessment of the speed of relief nasal congestion

**Table 4:** Clinical trials registered on the Clinicaltrials.gov database conducted on *Mentha* essential oil.

“*Mentha* Essential Oil” Clinical Trials	
Study Title	**Study of an Essential Oil and a Delmopinol Mouthrinse Effect on Dental Plaque Accumulation Index, Gingivitis Index and on Streptococcus Mutans, Lactobacillus, Aerobic and Anaerobic Oral Bacteria Colony Counts**
Condition	Gingivitis—Dental Plaque Accumulation
Status	Completed
Start and Completion dates	April 2007–April 2008
Study Type and Design	Interventional
	Allocation: Randomized
	Endpoint Classification: Efficacy Study
	Intervention Model: Parallel Assignment
	Masking: Open Label
	Primary Purpose: Prevention
Primary Outcome	Loe & Silness Gingival Index (1963)
	Quigley, Hein & Turesky Dental Plaque Index (1970)
Study Title	**Essential Oils Mouthrinse and Dental Floss, Comparison of Efficacy on Interproximal Gingivitis and Dental Plaque Accumulation**
Condition	Gingivitis Dental—Plaque Accumulation
Status	Completed
Start and Completion dates	September 2007–January 2008
Study Type and Design	Interventional
	Allocation: Randomized
	Endpoint Classification: Efficacy Study
	Intervention Model: Parallel Assignment
	Masking: Open Label
	Primary Purpose: Prevention
Primary Outcome	Lobene Gingival Index
	Saxton & Ouderaa Bleeding Index
	Quigley, Hein & Turesky Dental Plaque Index
Study Title	**Treatment of Acute Rhinosinusitis with Essential Oils of Aromatic Plants**
Condition	Rhinosinusitis
Status	Completed
Start and Completion dates	January 2008–May 2008
Study Type and Design	Interventional
	Allocation: Randomized
	Endpoint Classification: Efficacy Study
	Intervention Model: Parallel Assignment
	Masking: Double Blind (Subject, Investigator)
	Primary Purpose: Treatment
Primary Outcome	To demonstrate a relief in the nasal obstruction within the first 20 min after first administration of treatment with the spray
Study Title	**Treatment of Acute Pharyngo-Tonsillitis with Essential Oils of Aromatic Plants**
Condition	Viral Pharyngitis—Viral Tonsillitis
Status	Completed
Start and Completion dates	January 2008–May 2008
Study Type and Design	Interventional
	Allocation: Randomized
	Endpoint Classification: Efficacy Study
	Intervention Model: Parallel Assignment
	Masking: Double Blind (Subject, Investigator)
	Primary Purpose: Treatment
Primary Outcome	To demonstrate a throat pain relief within the first 20 min after first administration of treatment with the spray.
Study Title	**Treatment of Acute Tracheitis and Laryngitis with Essential Oils of Aromatic Plants**
Condition	Viral Laryngitis—Viral Tracheitis
Status	Completed
Start and Completion dates	January 2008–May 2008
Study Type and Design	Interventional
	Allocation: Randomized
	Endpoint Classification: Efficacy Study
	Intervention Model: Parallel Assignment
	Masking: Double Blind (Subject, Investigator)
	Primary Purpose: Treatment
Primary Outcome	To demonstrate a cough or hoarseness relief within the first 20 min after first administration of treatment with the spray
Study Title	**Dental Implants and Mouth Rinse**
Condition	Reduction in Bacterial Counts Through the Use of Mouthwash
Status	Recruiting
Start and Completion dates	November 2013–June 2016 (hypothesized)
Study Type and Design	Interventional
	Allocation: Randomized
	Endpoint Classification: Efficacy Study
	Intervention Model: Parallel Assignment
	Masking: Open Label
	Primary Purpose: Supportive Care
Primary Outcome	Oral Rinse Comparison
Study Title	**A Clinical Trial to Test the Effect of Marketed Mouth Rinses on Decreasing Plaque and Gum Inflammation**
Condition	Plaque—Gingivitis
Status	Completed
Start and Completion dates	August 2014–September 2014
Study Type and Design	Interventional
	Allocation: Randomized
	Endpoint Classification: Safety/Efficacy Study
	Intervention Model: Parallel Assignment
	Masking: Single Blind (Investigator)
Primary Outcome	Whole Mouth Mean Modified Gingival Index (MGI) at Week 3
	Whole Mouth Mean Plaque Index (PI) at Week 3
Study Title	**Freeze Remedy to Alleviate Morning Sickness**
Condition	Morning Sickness
Status	Recruiting
Start and Completion dates	August 2014–February 2015 (hypothesized)
Study Type and Design	Interventional
	Allocation: Randomized
	Endpoint Classification: Efficacy Study
	Intervention Model: Parallel Assignment
	Masking: Double Blind (Subject, Caregiver)
	Primary Purpose: Treatment
Primary Outcome	Improvement of Nausea and Vomiting of Pregnancy (NVP)

**Table 5:** Clinical trials registered on the Clinicaltrials.gov database conducted on *Citrus* essential oil.

“*Citrus* Essential Oil” Clinical Trials	
Study Title	**The Effects of Smell on Mood and Physical Responses**
Condition	Stress—Anxiety—Depression
Status	Completed
Start and Completion dates	August 2015–March 2016
Study Type and Design	Interventional
	Allocation: Randomized
	Endpoint Classification: Safety/Efficacy Study
	Intervention Model: Crossover Assignment
	Masking: Double Blind (Subject, Investigator)
	Primary Purpose: Treatment
Primary Outcome	Cortisol and Catecholamine Production; Immune Function; Skin Barrier Repair; Delayed Hypersensitivity to Candida (DTH)
Study Title	**Impact of AV2 Antiviral Drug on the Treatment of HPV-associated Lesions of the Uterine Cervix (KINVAV)**
Condition	Uterine Cervical Dysplasia—Papillomavirus Infections
Status	Recruiting
Start and Completion dates	January 2015–June 2016
Study Type and Design	Interventional
	Allocation: Randomized
	Endpoint Classification: Efficacy Study
	Intervention Model: Single Group Assignment
	Masking: Double Blind (Subject, Caregiver, Investigator, Outcomes Assessor)
	Primary Purpose: Treatment
Primary Outcome	Change of lesions (lesion size)
Study Title	**Aromatherapy for Chemotherapy-induced Symptoms**
Condition	Chemotherapy Symptoms
Status	Recruiting
Start and Completion dates	January 2016–July 2017
Study Type and Design	Interventional
	Allocation: Randomized
	Endpoint Classification: Efficacy Study
	Intervention Model: Parallel Assignment
	Masking: Double Blind (Subject, Outcomes Assessor)
	Primary Purpose: Treatment
Primary Outcome	Mean change in severity of symptoms
Study Title	**Limonene Study in Women with Breast Cancer**
Condition	Breast Cancer
Status	Completed
Start and Completion dates	August 2009–March 2011
Study Type and Design	Interventional
	Endpoint Classification: Pharmacokinetics/Dynamics Study
	Intervention Model: Single Group Assignment
	Masking: Open Label
	Primary Purpose: Prevention
Primary Outcome	Breast tissue limonene level
Study Title	**Does Massage with or without Aromatherapy Reduce Infant’s Distress? (aromatherapy)**
Condition	Distress
Status	Unknown
Start and Completion dates	January 2008–December 2009
Study Type and Design	Interventional
	Allocation: Randomized
	Endpoint Classification: Efficacy Study
	Intervention Model: Factorial Assignment
	Masking: Single Blind (Outcomes Assessor)
	Primary Purpose: Supportive Care
Primary Outcome	Level of COMFORT with videotaped COMFORT behavior scale
Study Title	**Freeze Remedy to Alleviate Morning Sickness**
Condition	Morning Sickness
Status	Recruiting
Start and Completion dates	August 2012–February 2015 (hypothesized)
Study Type and Design	Interventional
	Allocation: Randomized
	Endpoint Classification: Efficacy Study
	Intervention Model: Parallel Assignment
	Masking: Double Blind (Subject, Caregiver)
	Primary Purpose: Treatment
Primary Outcome	Improvement of Nausea and Vomiting in Pregnancy
